# Supplementary material for: Controlling Expansion and Cardiomyogenic Differentiation of Human Pluripotent Stem Cells in Scalable Suspension Culture
Source: Stem Cell Reports. 2014 Oct 30;3(6):1132–46. doi: 10.1016/j.stemcr.2014.09.017 (PMC4264033; doi:10.1016/j.stemcr.2014.09.017)
Supplement: Document S1. Supplemental Experimental Procedures and Figures S1–S5 [file mmc1.pdf]

Stem Cell Reports, Volume *3*

Supplemental Information

Controlling Expansion and Cardiomyogenic Differentiation of Human Pluripotent Stem Cells in Scalable Suspension Culture

Henning Kempf, Ruth Olmer, Christina Kropp, Michael Rückert, Monica Jara-Avaca, Diana Robles-Diaz, Annika Franke, David A. Elliott, Daniel Wojciechowski, Martin Fischer, Angelica Roa Lara, George Kensah, Ina Gruh, Axel Haverich, Ulrich Martin, and Robert Zweigerdt

Supplemental Figures

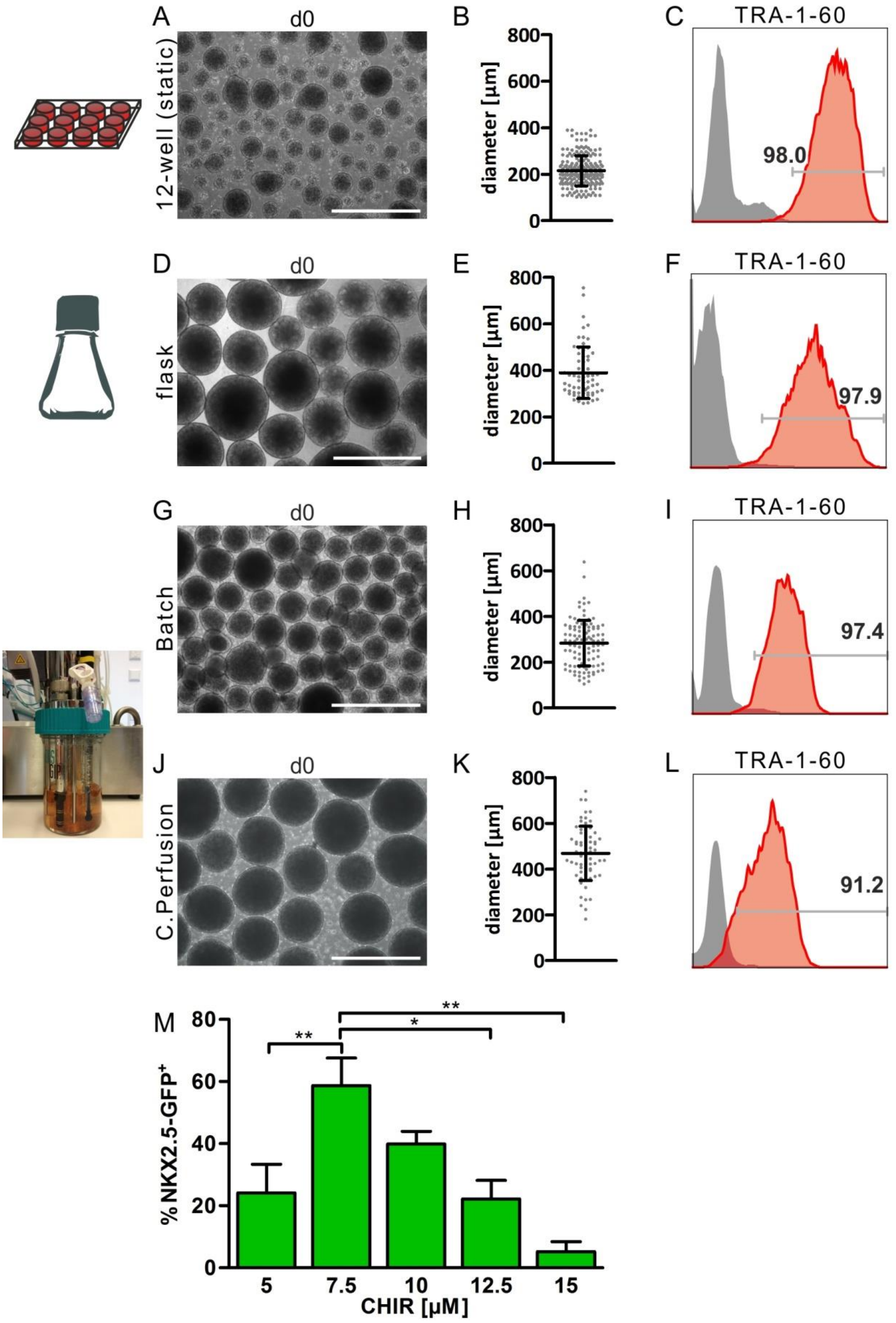

Figure S1. Characterization of aggregates before differentiation (day 0) generated in respective culture platforms, related to Figure 1, 2 and 3. Static culture **(A-C)**, agitated Erlenmeyer flask **(D-F)** and stirred bioreactors generated either by Batch **(G-I)** or C-Perfusion **(J-L)**, respectively. Representative microscopic images (left), quantification of aggregate diameters (middle; mean±SD) and flow cytometry data for TRA-1-60 positivity (right) are shown. Staining for TRA-1-60 (red) and respective IgM isotype controls (grey) are shown. Scale bars: 1 mm. **(M)** A similar pattern of NKX2.5-GFP was obtained using IWR1 instead of IWP2 and confirm 7.5 µM CHIR to induce highest level of GFP as quantified by flow cytometry on d10 of differentiation (n=3 independent experiments; mean±SEM).

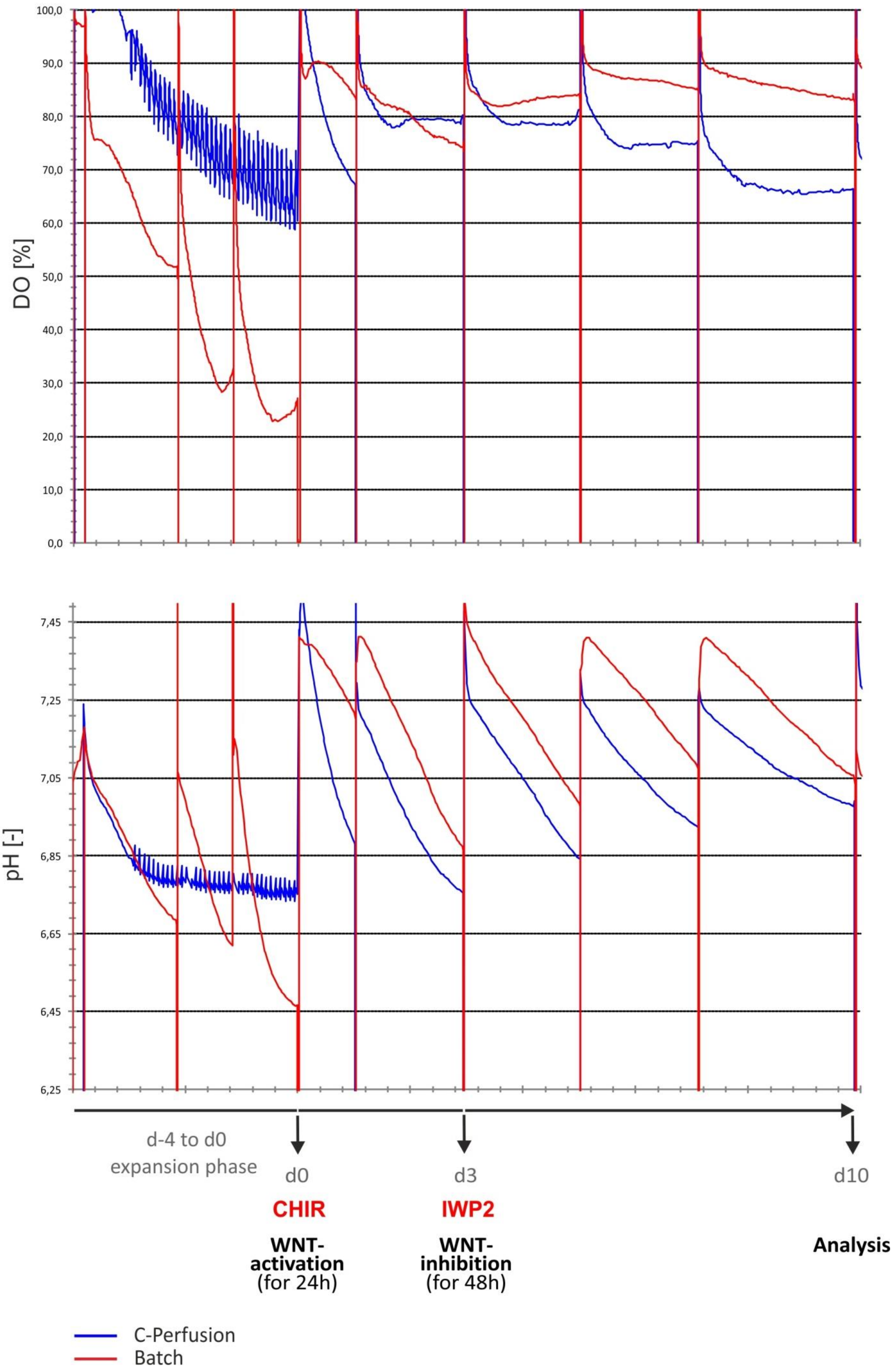

Figure S2. Online analysis of dissolved oxygen (DO) and pH during cell expansion and differentiation in stirred bioreactors, related to Figure 3. Representative DO levels (upper panel) and pH curves (lower panel) over time during the expansion phase (d-4 to d0) at C-Perfusion (blue line) or Batch feeding (red line) conditions and subsequent differentiation (d0 to d10) of respective cells.

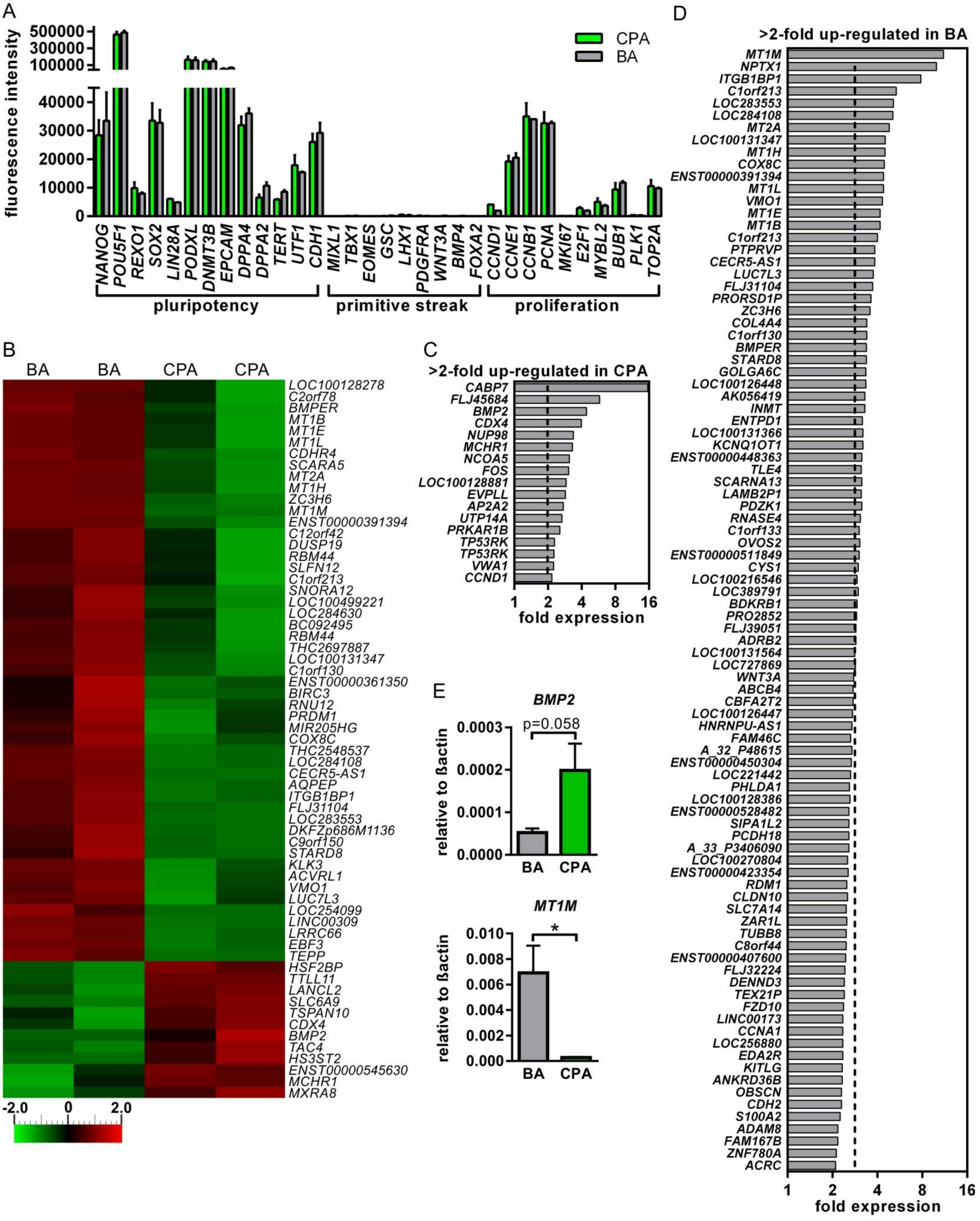

Figure S3. Microarray analysis comparing gene expression of bioreactor-derived aggregates (CPA vs. BA) prior to differentiation (day 0), related to Figure 3. (A) Absolute processed fluorescence intensity of markers for pluripotency, primitive streak formation and cell proliferation. **(B)** Heatmap displaying differentially expressed genes between BAs and CPAs detected by two group comparison using Qlucore Omics Explorer 3.0 ($p \leq 0.125$; $\sigma/\sigma_{max}=0.35$). **(C, D)** Up-regulated genes in CPA and BA, respectively, which were identified using the RCUTAS filter tool (Intensity threshold 50; fold change cut-off 2.0). For further details see the experimental section. **(E)** qPCR of *BMP2* (upper panel) and *MT1M* (lower panel) confirming the results of microarray analysis (n = 3 of independent bioreactor runs; mean±SEM).

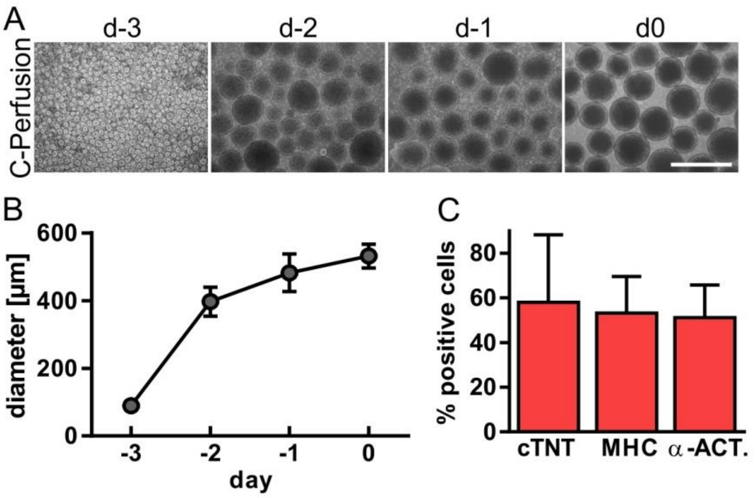

Figure S4. Characterization of Cyclic Perfusion bioreactor runs using the HSC_F1285T_iPS2 line confirms the robustness of the differentiation, related to Figure 4. (A) Representative images of aggregate formation in stirred bioreactors during C-Perfusion. Scale bar: 1mm **(B)** Quantification of aggregate diameters revealed formation of large spheres already 48h after culture inoculation (n=3 independent bioreactor runs, mean±SEM). **(C)** Differentiation efficiencies on d10 of differentiation (n=2 for cTNT, n=3 for MHC and α-ACTININ of independent bioreactor expansions; mean±SEM).

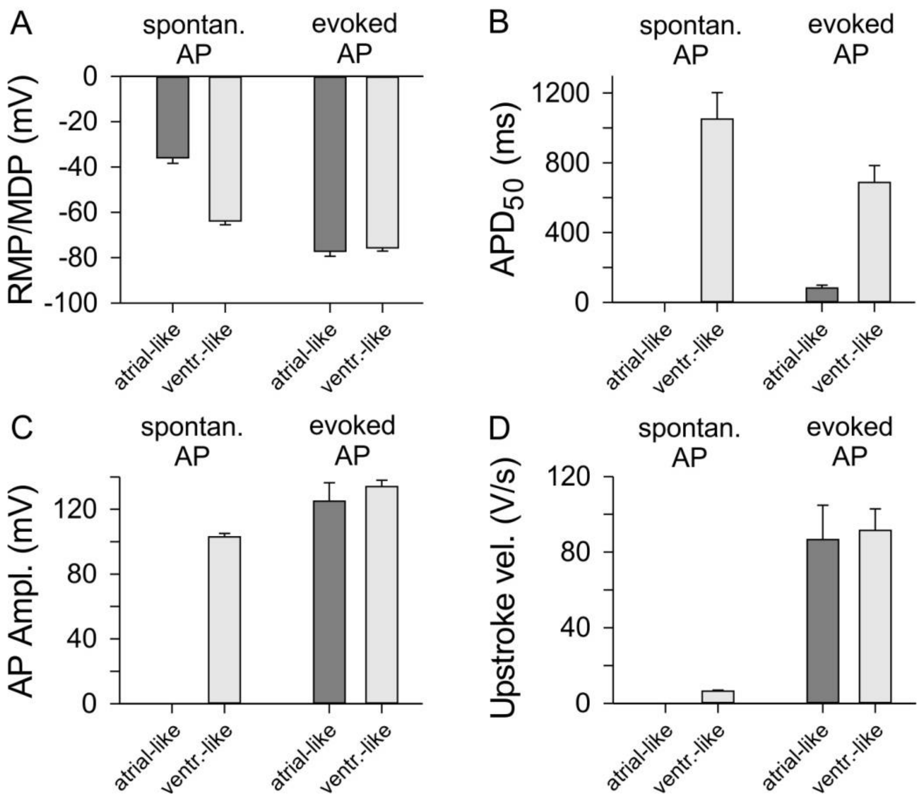

Figure S5. Electrophysiological properties of atrial-like (n=6) and ventricular-like (n=35) cardiomyocytes, related to Figure 5. Spontaneous action potentials (AP) are compared to APs evoked by intracellular injection of short depolarizing current steps (0.2 - 1.5 nA, 1 ms) after hyperpolarization of the plasma membrane to physiological resting potentials. **(A)** Atrial-like cells displayed small resting membrane potentials (RMP) and remained inactive without electrical stimulation. In contrast, 31 out of 35 ventricular-like cells were spontaneously active with a maximum diastolic potential (MDP) around -65 mV. Spontaneous activity declined with hyperpolarization to physiological resting potentials. **(B)** Evoked action potentials of atrial-like cells were shorter than those of ventricular-like cells (APD50: AP duration at 50% repolarization), whereas AP amplitudes and upstroke velocities were comparable **(C, D)**. Note that hyperpolarization to physiological resting conditions dramatically increased upstroke velocities of ventricular-like action potentials. Data represent mean±SEM.

Supplemental movie 1, related to Figure 3: Light and florescence microscopy of beating EBs of a CPA-based differentiation of the HES3 cell line.

Supplemental movie 2, related to Figure S4: Light microscopy of beating EBs of a CPA-based differentiation of the hHSC_1285T_iPS2 cell line.

Supplemental Experimental Procedures

Bioreactor calibration

Two-point calibration was applied for pH-probes. pO_2-probe calibration was conducted under process conditions: headspace-gassing with 3 L/h air/CO_2 (5%), 70 rpm stirring with 8 blade-pitched impeller, 37°C, 100 mL mTeSR1; reaching stable pO_2-values slope calibration was performed. For inoculation, 25 mL single cell suspension was added to achieve $5x10^5$ cells/mL in the final 125 mL culture volume.

Bioartificial cardiac tissue (BCT)

BCTs were prepared as described earlier (Kensah, 2013). In brief: ~60–80 bioreactor-derived EBs alone (w/o HFF) or with $1x10^5$ mitotically-inactivated human foreskin fibroblasts (w HFF) were mixed with a 0.8 mg/mL rat tail collagen I solution (R&D Systems) containing 10% matrigel (BD Biosciences) and poured into silicone molds for solidification in ~220 µL per tissue construct. Aiming at maturation and CM alignment, constructs were cultured and subjected to growing incremental static stretch (400 µm on d7, d11, d15, and d19) in a custom-made system (Kensah, 2011; Kensah, 2013). On d21 NKX2.5-GFP fluorescence was microscopically monitored to estimate group-dependent CM content (n=4 w/o HFF, n=5 w HFF). Force measurement: BCTs (n=3 w/o HFF, n=5 w HFF) were stretched by 200 µm increments allowing for preload-adjustment at each step and BCTs were electrically paced (25 V, 5 ms) five-times. The mean value of contraction force at each respective preload-position was recorded. L_{max} describes the length, i.e. preload position of individual tissue constructs, where maximum contraction forces were observed. Passive force determination: comparison of force sensor values at starting position versus values at respective preload step resulted in ΔmN. Additionally, passive force values at L_{max} for individual BCTs were determined.

Multielectrode Array (MEA)

Multielectrode array was performed as described previously (Wunderlich, 2012). In brief, bioreactor-derived EBs at d8-10 were dissociated and seeded at $0.5\text{-}1x10^6$ cells/array (60MEA200/30iR-Ti-gr, Multi Channel Systems; 0.1% gelatin, 4 µg/mL fibronectin-coated) in 1 mL IMDM (Invitrogen) containing 20% FCS (HyClone), 100 µM β-mercaptoethanol, 1% nonessential amino acids, 1% penicillin/streptomycin (Biochrom) and 10 µM Y-27632. All tested substances were purchased from Sigma-Aldrich, diluted in ultrapure water and added at concentrations as indicated. Recordings were performed and processed in MC-Rack (Multi Channel Systems) and visualized using GraphPad Prism.

Electrophysiology

CPA-derived cardiomyocytes were plated on gelatin/fibronectin-coated cover slips at differentiation day 10-13 and standard whole-cell patch clamp recordings were performed between day 19 and 27 of differentiation using an Axopatch 200B amplifier (Molecular Devices, Sunnyvale, CA). Pipettes were pulled from borosilicate glass (Harvard Apparatus, Holliston, MA) with resistances between 2 MΩ and 6 MΩ. The extracellular Thyrode solution contained (in mM): NaCl (140), KCl (5.4), CaCl2 (1.8), MgCl2 (1), HEPES (10), glucose (10), pH 7.4 (adjusted with NaOH). The intracellular solution contained (in mM): K-gluconate (120), MgCl2 (1), Mg-ATP (3), EGTA (10), HEPES (10), pH 7.2 (adjusted with KOH). Agar bridges were used to connect the amplifier and the pipette solution. Results were corrected for junction potentials calculated using the JPCalc software (Dr. P. Barry, University of South Wales, Sydney, Australia (Barry, 1994). Membrane potentials and spontaneous action potentials of single cardiomyocytes were recorded in the current clamp mode. Input resistances were determined from responses to small current steps and ranged from 500 MΩ to 5 GΩ. Cells with lower resistances were considered as leaky cells and rejected from evaluation. Cardiomyocytes were classified as atrial-like or ventricular-like based on the shape of their action potentials (AP). Resting membrane potential (RMP) or maximum diastolic potential (MDP), AP amplitude, AP duration at 50% repolarization (APD50) and upstroke velocity were determined. Cells with short plateau phase (< 200 ms) were classified as atrial-like cardiomyocytes. Electrophysiological properties were obtained from spontaneous firing and from evoked action potentials while cells were hyperpolarized to physiological resting potentials (70-80 mV). In the latter case action potentials were elicited by short depolarizing current steps (200 pA to 1.5 nA; 1 ms). Data were analyzed using Clampfit software (Axon Instruments, Union City, CA) and are presented as means±SEM.

Flow cytometry

For intracellular staining, $1.5x10^5$ cells were fixed/permeabilized according to manufacturer's instructions (Fix&Perm-kit; An der Grub). Antibodies specific to cardiac troponin T (1:200, clone 13-11, Thermo Scientific), α-ACTININ (1:800, clone EA-53, Sigma-Aldrich), myosin heavy chain (1:25, Hybridoma Bank; 1:2000, clone NOQ7.5.4D, Sigma-Aldrich), SIRPα (clone SE5A5; Biolegend), NKX2.5 (1:200; clone H-114; Santa Cruz) and respective isotype controls (Dako) were detected using appropriate Cy3-/Cy5-conjugated antibodies (1:200; Jackson Immunoresearch Laboratories). For surface staining, $1.5x10^6$ cells were incubated with anti-TRA-1-60-specific (1:100; mouse IgM, Abcam) and corresponding IgM isotype control (Dako) antibodies for 30 min at 4°C. After washing, signals were detected using Cy3-labeled donkey anti-mouse IgM (1:200; Jackson Immunoresearch Laboratories) on the Accuri C6 flow cytometer (BD Biosciences). Data were analyzed using FlowJo (Treestar).

Immunofluorescent staining

Dissociated cells were seeded on 0.1% gelatin-coated culture plates and fixed after 2 days with 4% paraformaldehyde, 15 min, RT. 10 µm cryosections were generated from liquid nitrogen-frozen aggregates/EBs embedded in Tissue-Tek (Sakura-Finetek) on HM 560 cryotome (Thermo Scientific). After blocking by Tris-buffered saline (5% donkey serum, 0.25% Triton X-100) cells or cryosections were incubated with primary/secondary antibodies listed in 'Flow cytometry', respectively. Nuclei were DAPI-stained and samples were analyzed using the Axio Observer A1 (Zeiss) or a DM IRB/TCS SP2 confocal microscope system (Leica).

Quantitative real-time PCR

Total RNA was prepared using RNeasy-Kit (Macherey-Nagel), reverse-transcribed with RevertAid^{TM} H-Minus (Fermentas, Thermo Scientific) using random primers. qRT-PCR was performed using the Solaris qPCR Expression Assays (Thermo Scientific); expression levels of target genes were normalized to β-Actin levels.

Microarray analysis

The microarray has been performed by use of a refined version of the Whole Human Genome Oligo Microarray 4x44K v2 (Design ID 026652, Agilent Technologies), called '026652AsQuintuplicatesOn180k' developed in the Research Core Unit Transcriptomics of Hannover Medical School (design ID 054261) covering roughly 26000 human transcripts. Microarray design was defined at Agilent's eArray portal using an mRNA expression 4x180k design format as template. All non-control probes of design ID 026652 have been determined to be printed five times onto one 180k Microarray (on-chip quintuplicates). Control probes required for proper Feature Extraction software algorithms were determined and placed automatically by eArray using recommended default settings. Synthesis of Cy3-labeled cRNA was performed with the 'Quick Amp Labeling kit, one color' (#5190-0442, Agilent Technologies) according to the manufacturer's recommendations. cRNA fragmentation, hybridization and washing steps were also carried-out exactly as recommended in the 'One-Color Microarray-Based Gene Expression Analysis Protocol V5.7'. 1650ng of each labeled cRNA population were used for hybridization. Slides were scanned on the Agilent Micro Array Scanner G2565CA (pixel resolution 3 µm, bit depth 20). Data extraction was performed with the 'Feature Extraction Software V10.7.3.1' using a modified version of the recommended default extraction protocol file 'GE1_107_Sep09.xml' in which the minimal number of replicates to calculate Population Outliers was set to 5.

To identify >2-fold up-regulated genes the processed intensity values of the green channel, 'gProcessedSignal' (gPS) were normalized by global linear scaling: All gPS values of one

sample were multiplied by an array-specific scaling factor. This factor was calculated by dividing a ‘reference 75th Percentile value’ (set as 1500 for the whole series) by the 75th Percentile value of the particular Microarray (‘Array I’ in the formula shown below). Accordingly, normalized gPS values for all samples (microarray data sets) were calculated by the following formula:

$$normalized\ gPS_{Array\ i} = gPS_{Array\ i} \times (1500 / 75^{th}\ Percentile_{Array\ i})$$

Measurements of on-chip replicates (quintuplicates) were averaged using the arithmetic mean of normalized gPS values to retrieve one resulting value per probe and sample. Features that were marked as Outliers by the Feature Extraction software were excluded from averaging.

A lower intensity threshold (surrogate value) was defined as 1% of the reference 75th Percentile value (= 15). All of those normalized gPS values that fell below this intensity border, were substituted by the respective surrogate value of 15. Differentially expressed genes were identified by filtering >2-fold up-regulated genes (Intensity threshold 50; fold change cutoff 2.0) applying the RCUTAS filter tool (Research Core Unit Transcriptomics of Hannover Medical School). Results were ranked by fold change and displayed in a diagram.

Alternatively, extracted data were loaded into Qlucore Omics Explorer 3.0 (Qlucore AB, Lund, Sweden) applying default settings including normalization and baseline transformation. A two group comparison ($p \leq 0.125$; $\sigma / \sigma_{max} = 0.35$) was performed and visualized in a hierarchically clustered heatmap. Microarray data are available in the ArrayExpress database (www.ebi.ac.uk/arrayexpress) under accession number E-MTAB-2814.

Supplemental References

Barry, P. H. (1994). JPCalc, a software package for calculating liquid junction potential corrections in patch-clamp, intracellular, epithelial and bilayer measurements and for correcting junction potential measurements. J Neurosci Methods 51, 107-116.

Kensah, G., et al. (2011). A novel miniaturized multimodal bioreactor for continuous in situ assessment of bioartificial cardiac tissue during stimulation and maturation. Tissue engineering. Part C, Methods 17, 463-473.

Kensah, G., et al. (2013). Murine and human pluripotent stem cell-derived cardiac bodies form contractile myocardial tissue in vitro. Eur Heart J 34, 1134-1146.

Wunderlich, S., et al. (2012). Induction of pluripotent stem cells from a cynomolgus monkey using a polycistronic simian immunodeficiency virus-based vector, differentiation toward functional cardiomyocytes, and generation of stably expressing reporter lines. Cell Reprogram 14, 471-484.
