# Supplementary material for: Controlling Expansion and Cardiomyogenic Differentiation of Human Pluripotent Stem Cells in Scalable Suspension Culture
Source: Stem Cell Reports. 2014 Oct 30;3(6):1132–46. doi: 10.1016/j.stemcr.2014.09.017 (PMC4264033; doi:10.1016/j.stemcr.2014.09.017)
Supplement: Document S2. Article plus Supplemental Information [file mmc4.pdf]

Stem Cell Reports
Resource
OPEN ACCESS

Controlling Expansion and Cardiomyogenic Differentiation of Human Pluripotent Stem Cells in Scalable Suspension Culture

Henning Kempf,[1,2,6] Ruth Olmer,[1,2,3,6] Christina Kropp,[1,2] Michael Rückert,[1,2] Monica Jara-Avaca,[1,2] Diana Robles-Diaz,[1,2] Annika Franke,[1,2] David A. Elliott,[4] Daniel Wojciechowski,[5] Martin Fischer,[5] Angelica Roa Lara,[1,2,7] George Kensah,[1,2,8] Ina Gruh,[1,2] Axel Haverich,[1,2] Ulrich Martin,[1,2,3] and Robert Zweigerdt[1,2,*]

[1]Leibniz Research Laboratories for Biotechnology and Artificial Organs (LEBAO), Department of Cardiothoracic, Transplantation, and Vascular Surgery (HTTG), Hannover Medical School, Carl-Neuberg-Straβe 1, 30625 Hannover, Germany
[2]REBIRTH-Cluster of Excellence, Hannover Medical School, Carl-Neuberg-Straβe 1, 30625 Hannover, Germany
[3]Member of the Biomedical Research in Endstage and Obstructive Lung Disease Hannover (BREATH), Member of the German Center for Lung Research (DZL), 30625 Hannover, Germany
[4]Murdoch Childrens Research Institute, The Royal Children's Hospital, Flemington Road, Parkville, VIC 3052, Australia
[5]Institute for Neurophysiology, Hannover Medical School, Carl-Neuberg-Straβe 1, 30625 Hannover, Germany
[6]Co-first author
[7]Present address: Institute of Pharmacology, University Medical Center, Georg-August University Göttingen, Robert-Koch-Straβe 40, 37075 Göttingen, Germany
[8]Present address: Department of Cardiothoracic Surgery, Otto-von-Guericke University Magdeburg, Leipziger Straβe 44, 39120 Magdeburg, Germany
*Correspondence: zweigerdt.robert@mh-hannover.de
http://dx.doi.org/10.1016/j.stemcr.2014.09.017
This is an open access article under the CC BY license (http://creativecommons.org/licenses/by/3.0/).

SUMMARY

To harness the potential of human pluripotent stem cells (hPSCs), an abundant supply of their progenies is required. Here, hPSC expansion as matrix-independent aggregates in suspension culture was combined with cardiomyogenic differentiation using chemical Wnt pathway modulators. A multiwell screen was scaled up to stirred Erlenmeyer flasks and subsequently to tank bioreactors, applying controlled feeding strategies (batch and cyclic perfusion). Cardiomyogenesis was sensitive to the GSK3 inhibitor CHIR99021 concentration, whereas the aggregate size was no prevailing factor across culture platforms. However, in bioreactors, the pattern of aggregate formation in the expansion phase dominated subsequent differentiation. Global profiling revealed a culture-dependent expression of BMP agonists/antagonists, suggesting their decisive role in cell-fate determination. Furthermore, metallothionein was discovered as a potentially stress-related marker in hPSCs. In 100 ml bioreactors, the production of 40 million predominantly ventricular-like cardiomyocytes (up to 85% purity) was enabled that were directly applicable to bioartificial cardiac tissue formation.

INTRODUCTION

Cardiovascular disorders can induce severe, progressive loss of contractile heart muscle tissue, including billions of cardiomyocytes (CMs). Because of the low regenerative capacity of the heart, this can ultimately lead to heart failure with very limited treatment options available at present (Donndorf et al., 2013). Given their proliferation and differentiation potential, human pluripotent stem cells (hPSCs), including embryonic stem cells (hESCs) and induced pluripotent stem cells (hiPSCs), are an attractive cell source for the mass generation of lineage- and (potentially) patient-specific progenies, including bona fide CMs (Laflamme and Murry, 2011). This possibility opens new avenues for the development of regenerative cell therapies and more specific drug discovery assays. Therapeutic and industrial applications of hPSCs, however, will require large cell quantities to be generated under highly robust, well-defined, and economically viable conditions (Zweigerdt, 2009).

It was recently shown that hPSCs can be expanded as cell-only aggregates in serum-free suspension culture irrespective of matrix supplementation (Amit et al., 2011; Olmer et al., 2010; Singh et al., 2010), which is mandatory for conventional surface-attached propagation of hPSCs. In contrast to matrix-attached 2D conditions, suspension culture (3D) provides a straightforward strategy for process upscaling, including cell cultivation in stirred tank bioreactors (Couture, 2010). Stirred tank reactors represent a universal, well-established vessel type for the production of recombinant proteins in industrial biotechnology (Carrondo et al., 2012) and allow for cost-effective, multiparametric monitoring and optimization of mammalian cell culture processes (Bulnes-Abundis et al., 2013). Once established, relative linear process upscaling is feasible since reactors from 0.1 to >1.000 l culture scale are available. However, the application of stirred bioreactors to hPSC expansion and their differentiation is still in its infancy.

Single cell-based inoculation of suspension cultures establishes a well-controlled starting point at every passage (Zweigerdt et al., 2011). The inoculation density as well as the physical properties of the culture system (such as the reactor design and the stirring speed) can then be

used to control formation of PSC aggregates and their subsequent growth (Olmer et al., 2012; Schroeder et al., 2005). Importantly, when utilizing appropriate media such as mTeSR, hPSCs remain pluripotent over multiple passages in aggregate culture (Olmer et al., 2010; Zweigerdt et al., 2011), thus providing the attractive option of directly switching from hPSC expansion to lineage-specific differentiation in a continuous suspension process.

Recent work has demonstrated that Wnt pathway modulation by small molecules is an efficient strategy for hPSC cardiomyogenic induction, resulting in ~60%–80% CMs content in defined media (Gonzalez et al., 2011; Lian et al., 2012; Minami et al., 2012). A common feature of these protocols is the activation of the Wnt pathway at early stages of differentiation by the GSK3 inhibitor CHIR99021 (CHIR) aiming at enhanced mesoderm induction. Following cues from developmental biology, Wnt pathway activity is then inhibited using inhibitors such as IWP (inhibitor of Wnt production) or IWR (inhibitor of Wnt response). This later step aims at specifying cardiac differentiation of the mesoderm-directed cells (Hudson et al., 2012; Lian et al., 2012; Ren et al., 2011; Willems et al., 2011). However, these protocols rely on confluent monolayer cultures limiting straightforward industrial scale production.

In this study, we aimed at directly combining hPSC expansion with cardiomyogenic differentiation in suspension culture. Taking advantage of a NKX2.5-GFP reporter line (Elliott et al., 2011), a multiwell screening assay was established to develop Wnt modulator-based CMs differentiation of hPSC aggregates in static suspension culture. By scaling up to rotated Erlenmeyer flasks and ultimately to fully equipped stirred tank bioreactors, we show the robustness of the method, as well as its applicability to dynamic suspension culture. The work provides insights on critical cellular and molecular process parameters and a straightforward strategy for the scalable mass production of CMs at up to 85% purity, which predominantly displayed ventricular-like action potentials (APs). Moreover, bioreactor-derived embryoid bodies (EBs) were directly applicable for the generation of human bioartificial cardiac tissue (BCT) (Kensah et al., 2011), a promising strategy for heart repair and novel in vitro drug discovery and drug safety assays.

RESULTS

Multiwell Screen Reveals Tight CHIR Concentration Dependence of Cardiomyogenic Differentiation in Suspension Culture

To establish combined hPSC expansion and cardiac differentiation, single cell-dissociated HES3 NKX2-5$^{eGFP/w}$ were seeded into 12-well dishes in mTeSR1 (Figure 1A). After 4 days, a reproducible pattern of floating aggregates (214 ± 65 μm average diameter; Figures S1A and S1B available online) formed comprising ~1 million cells per well (data not shown); ~98% TRA-1-60 positivity (Figure S1C) suggested maintenance of pluripotency in this culture format, in line with previous findings (Olmer et al., 2010; Zweigerdt et al., 2011).

mTeSR1 was replaced with differentiation medium (3 ml/well; ~0.33 million cells/ml) supplemented with 0–15 μM CHIR for 24 hr, followed by IWP2 supplementation on day 3 for 48 hr (Figure 1A). On day 10, fluorescence microscopy of floating EBs and flow cytometry revealed the highest content of 52% ± 7.3% NKX2.5-GFP-positive cells (GFP$^+$; indicative of CMs) at 7.5 μM CHIR (Figures 1B and 1C). The highest total cell count was also observed under these conditions (Figure 1D). Somewhat lower cell yields and GFP$^+$ cells were obtained at 5 μM CHIR, whereas significantly lower CMs induction was observed at 10 μM CHIR, which further declined in response to higher drug concentrations (Figures 1B–1D). In DMSO controls (0 μM CHIR), EBs disintegrated over time, suggesting loss of vital cells (Figure 1B).

Replacing IWP2 by the alternative Wnt pathway inhibitor IWR1 resulted in a shift toward reduced GFP$^+$ cells at 5 μM but elevated GFP$^+$ at 10 μM CHIR (Figure S1M). However, cardiomyogenic induction remained highest at 7.5 μM CHIR, resulting in 58.7% ± 8.8% GFP$^+$ irrespective of IWR1 or IWP2. Thus, IWP2 was maintained throughout further experiments.

The established protocol was robust in experimental repeats and confirmed by two human iPSC lines differentiated at 7.5 μM CHIR. Immunofluorescence and flow cytometry specific to cardiac troponin T (cTNT), sarcomeric α-actinin, myosin heavy chain (MHC), and NKX2.5, respectively, revealed the induction of up to ~60% CMs in these hiPSC lines (Figures 1E and 1F).

The Static Suspension Protocol Is Scalable to Rotated Erlenmeyer Flasks

Dynamic conditions can have a profound impact on stem cell culture and differentiation (Fridley et al., 2012). We thus tested whether the static 12-well format can be directly transferred to rotated Erlenmeyer flasks (Figure 2A).

In single cell-inoculated flasks, a relatively fast increase in aggregate size during the initial 48 hr was observed (data not shown) and aggregates with an average diameter of 389.4 ± 13.8 μm formed in 4 days, with ~98% of cells remaining TRA-1-60 positive (Figures S1D and S1F). Differentiation by 7.5 μM CHIR resulted in robust GFP expression and formation of contractile EBs (Figure 2B). CM induction was confirmed by immunohistochemistry specific to sarcomeric markers on EB-derived sections and dissociated cells (Figure 2C). On average, 55% ± 7.4% GFP$^+$ cells were obtained on days 7–10 (Figure 2D) with >60% positivity to

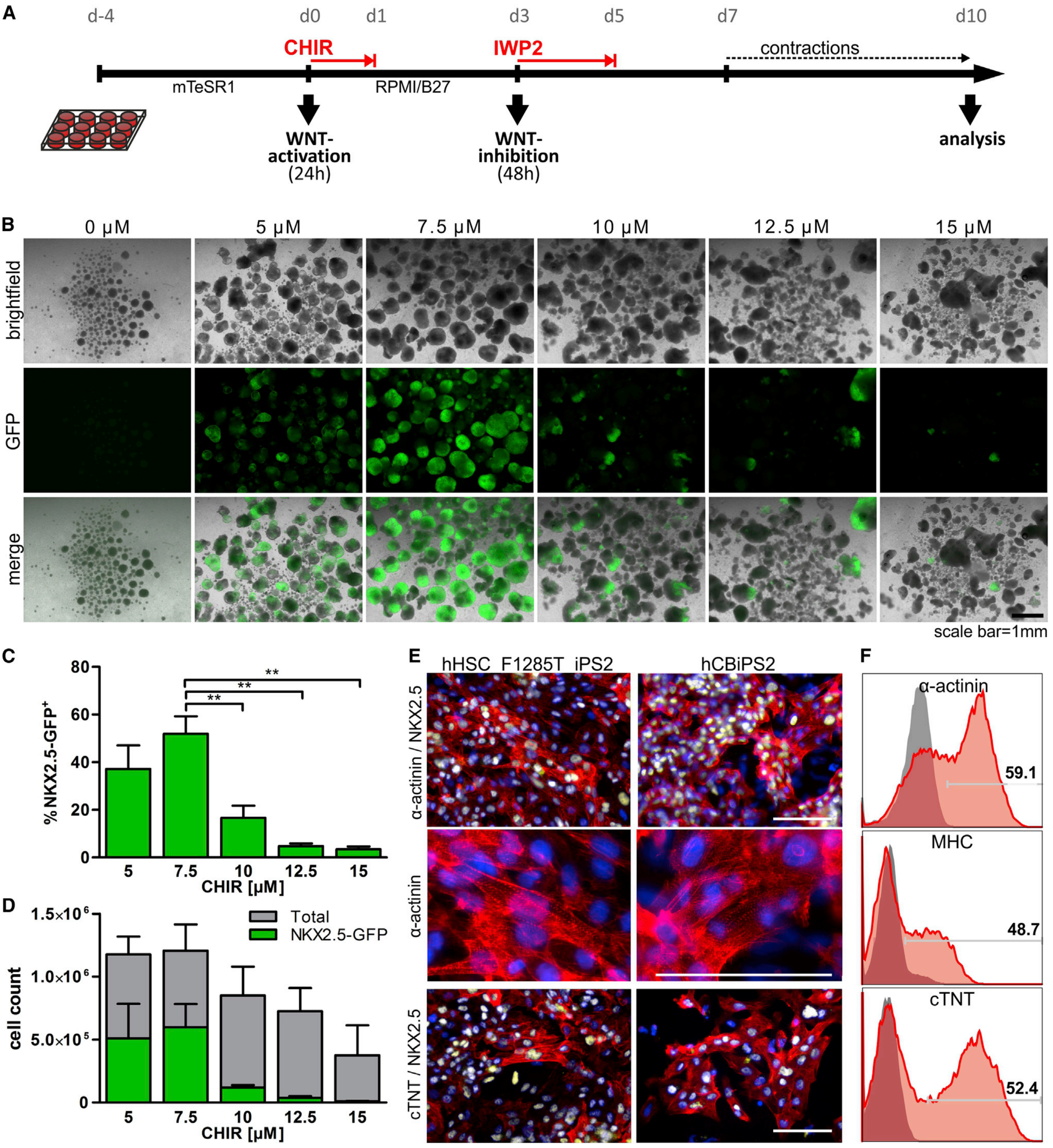

Figure 1. Efficient Cardiomyogenic Differentiation in Static Suspension Culture Works Only in a Tight Range of CHIR Concentration
(A) Scheme of the expansion/differentiation protocol in a 12-well format.
(B) Microscopic assessment of NKX2.5-GFP transgenic HES3 EBs on day 10 in response to the respective CHIR concentration supplemented for 24 hr at day 0.
(C) Flow cytometry on day 10 revealed the highest content of GFP$^+$ cells at 7.5 μM CHIR (n = 3 independent experiments, mean ± SEM).
(D) Bars represent the total cell count per well and respective GFP content (green), confirming 7.5 μM CHIR as the most efficient concentration for CM induction (n = 3 experiments, mean ± SEM).

(legend continued on next page)

cTNT and signal-regulatory protein alpha (SIRPα), respectively (Figure 2E).

Alternative Feeding Strategies Impact on Aggregate Formation in Stirred Bioreactors

To increase the production of CMs, we moved our combined expansion/differentiation protocol to 100 ml stirred bioreactors and examined two alternative expansion strategies. First, we attempted "batch-feeding" (batch), in which cells were allowed to form stirring-controlled aggregates for 48 hr before 100 ml mTeSR1 was exchanged daily. The strategy resulted in a relative linear increase of "batch-aggregates" (BAs) in size over time (Figures 3A and 3B), in line with published observations (Olmer et al., 2012).

The second strategy termed "cyclic perfusion feeding" (C-perfusion) aimed at more homogeneous culture conditions by continuous, automated medium exchange: at 24 hr after inoculation, stirring was paused for 10 min every 2 hr to let cell aggregates settle by gravity. About 7 ml of medium was automatically replaced at every cycle, resulting in a turnover of ~90 ml/day (approximately equivalent to the 100 ml daily medium throughput by batch). Online monitoring of metabolic activity by means of dissolved oxygen (DO) and pH revealed that both parameters remained more stable in C-perfusion compared with batch culture (Figure S2). The pattern of C-perfusion aggregate (CPA) formation was also different, as relatively large spheres readily formed at an early stage (Figures 3A and 3B), reminiscent of the Erlenmeyer flask approach. Such process-dependent aggregate patterns were reproducible in individual runs (Figure 3B), resulting in 283 ± 9.6 μm average diameter for BAs compared with 468 ± 14.4 μm for CPAs on day 0 (compare Figures S1G and S1H with Figures S1J and S1K). Notably, the pluripotency markers TRA-1-60 (Figures 1I and 1L), *NANOG*, and *OCT4* (quantitative RT-PCR [qRT-PCR]; Figure 3F) had similar expression profiles in both culture conditions.

Global gene expression analysis comparing the undifferentiated aggregates derived from batch and C-perfusion on day 0 revealed a highly equivalent expression signature of established pluripotency, as well as cell proliferation genes and primitive-streak markers (Figure S3A). To identify potential differences, array data were processed in a group comparison by the programs Qlucore Omics and RCUTAS, filtering for genes that were statistically significant and >2-fold regulated, respectively (see respective gene lists in Figures S3B–S3D). A set of 21 genes was found to be upregulated in BAs by both programs (Figure 3C). Notably, these included six members of the metallothionein family (*MT1M*, *MT2A*, *MT1H*, *MT1L*, *MT1E*, *MT1B*), known to bind metal ions (gene ontology categories: cellular response to zinc ion, metal ion, inorganic substances, negative regulation of growth). In CPAs, only three genes were found to be upregulated under the same comparative criteria: the TGF-beta superfamily member *BMP2,* the caudal type homeobox transcription factor (*CDX4*), and the melanin-concentrating hormone receptor 1 (*MCHR1*; Figure 3C). Exemplary verification of the array data confirmed upregulation of *BMP2* in CPAs and *MT1M* in Bas, as depicted in Figure S3E.

The Preceding Expansion Strategy Determines Differentiation Outcome in Bioreactors

Before inducing aggregate differentiation, cell numbers were determined. On average, batch expansion yielded ~79 million HES3-NKX2-5$^{eGFP/w}$ cells per bioreactor, whereas ~60 million cells were recovered by C-perfusion (n = 2 runs each; data not shown). Subsequently, aggregates were diluted in differentiation medium (7.5 μM CHIR) to establish ~33 million cells per 100 ml (~0.33 million cells/ml equivalent to 12-well cultures) per bioreactor. After this "equalization step," the same differentiation scheme (Figure 3D) was applied in stirred reactors irrespective of the preceding expansion strategy. Surprisingly, we found that differentiation runs (n = 3) initiated with BAs repeatedly failed to form contracting EBs and essentially no GFP$^+$ cells were detected (Figure 3E). In contrast, contracting EBs were observed upon CPAs differentiation, which was accompanied by GFP expression in all experimental repeats (n = 3; Figure 3E).

NANOG and *OCT4* expression was equivalent in both expansion approaches before differentiation (day 0; Figure 3F). However, after 24 hr of CHIR treatment (day 1), *NANOG* dropped substantially in BAs but remained almost unchanged in CPAs before downregulation occurred between days 1 and 3. A decline of *OCT4* expression was also delayed in CPAs (Figure 3F).

Regarding the metabolic activity, induction of differentiation of CPAs resulted in a steep DO drop from 100% to ~65% in 24 hr, whereas a moderate reduction to ~85% occurred in BA differentiation (exemplary depicted in Figure S2). Accordingly, the pH dropped from 7.5 to 6.9 in CPA but only to 7.2 in BA differentiation. Thus, although differentiation cultures were initiated at equivalent cell

(E and F) Differentiation of the hiPSC lines hHSC_F1285T_iPS2 and hCBiPS2 at 7.5 μM CHIR confirmed the robustness of the established protocol, as shown by microscopic images of plated cells derived from day 10–13 EBs (E; NKX2.5 in yellow, α-actinin and cTNT in red, DAPI in blue; scale bars represent 100 μm) and flow cytometry of d10 EB-derived CBiPS2 cells specific to respective sarcomeric proteins. (F) Isotype controls in gray.
See also Figure S1.

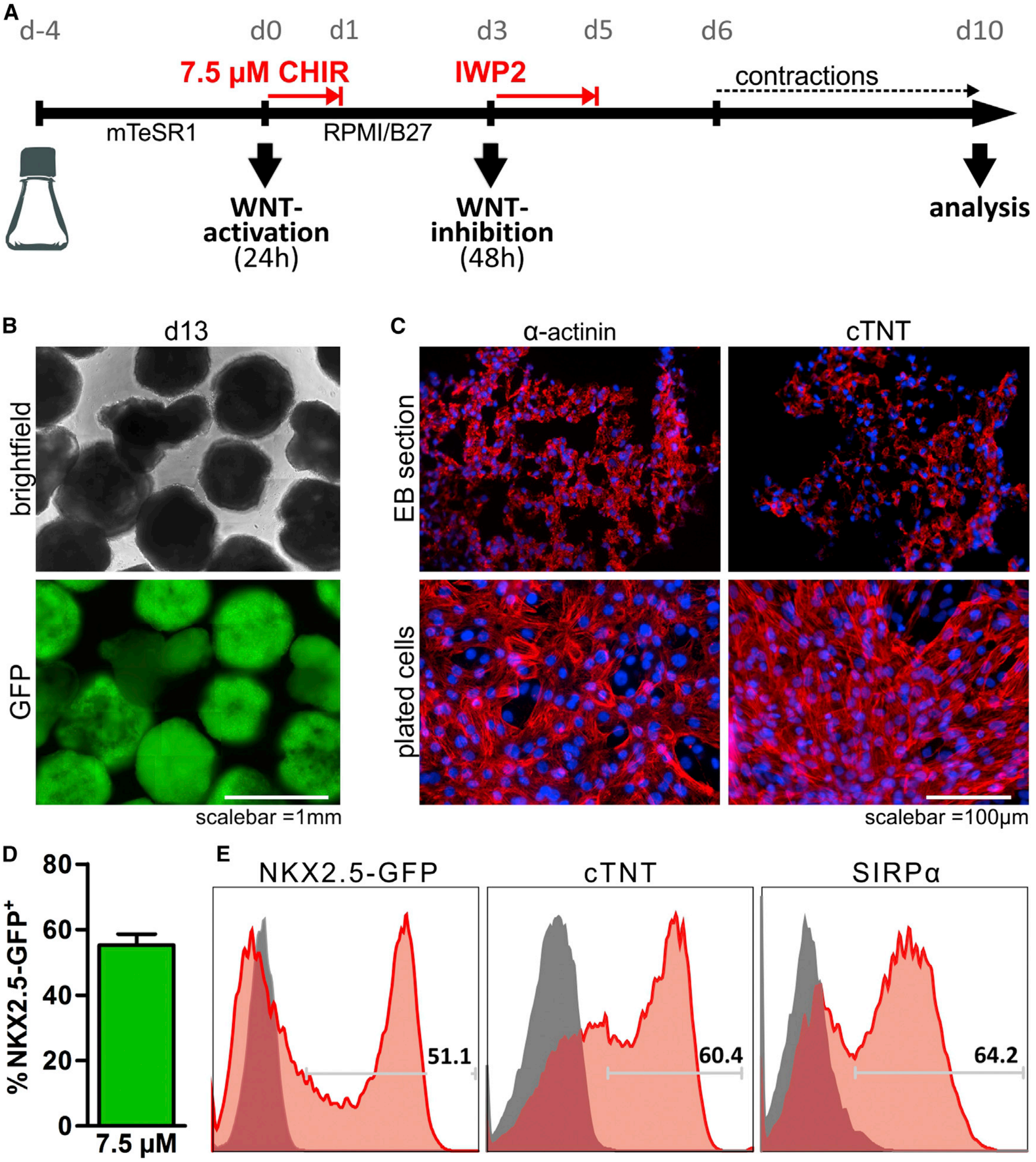

Figure 2. Protocol Scale Up to Rotated Erlenmeyer Flasks

(A) Culture scheme in flasks rotated at 75 rpm.

(B) Microscopic assessment of NKX2.5-GFP expression in EBs on day 13 of differentiation in response to 7.5 µM CHIR.

(C) Immunofluorescent staining specific to α-actinin and cTNT on EB sections (top) and dissociated/plated cells derived thereof (bottom).

(D) Flow cytometry on day 7–10 revealed ~55% of GFP$^+$ (n = 5 of four independent experiments; mean ± SEM).

(E) Representative flow cytometry histograms of day 10 EB-derived cells.

See also Figure S1.

densities and performed under the same conditions, the overall metabolic activity was substantially higher in CPAs in the presence of CHIR and remained higher compared with differentiation processes of BAs during IWP2 treatment and thereafter (Figure S2).

Robust upregulation of primitive streak markers Brachyury T (*T*) (Gadue et al., 2006) and *MIXL1* (Davis et al., 2008) was observed after CHIR treatment (day 1), irrespective of aggregate origin, suggesting equivalent progression of early differentiation in both conditions (Figure 3F). A

differential pattern was observed for *MESP1*, a key regulator of mesoderm and cardiovascular cell-fate determination (Bondue et al., 2008; David et al., 2008), and *TBX3*, promoting early lineage (Esmailpour and Huang, 2012), mesendoderm (Weidgang et al., 2013), and CM specification (Hoogaars et al., 2007). Upregulation of both genes was more rapid in differentiating BAs (which ultimately failed to form CMs) preceding expression in CPAs by 24–48 hr (Figure 3F). Subsequently, upregulation of the early cardiac progenitor markers *ISL1* (Lui et al., 2013) and *NKX2.5* (Elliott et al., 2011) was almost exclusively observed in CPA differentiations, consistent with the GFP expression. On the other hand, *GATA4* expression, a gene involved in cardiac mesoderm (Sepulveda et al., 1998), as well as endodermal lineage specification (Agarwal et al., 2008), was highly equivalent in cells from both expansion strategies (Figure 3F).

Directed Differentiation Enabled up to 85% CM Induction and Generation of 40 Million CMs in 100 ml Scale

Focusing on CM formation, CPA-based differentiation processes were analyzed in more detail. Upregulation of cTNT (*TNNT2*) and α-MHC (*MYH6*) was observed by qRT-PCR from day 5 onward (Figure 4A) in line with contracting EBs from days 6 and 7 onward (data not shown). Interestingly, α-MHC expression dropped when comparing day 7 versus day 10 samples, whereas β-MHC (*MYH7*), a marker of CMs maturation (Lundy et al., 2013), showed robust upregulation (Figure 4A).

At endpoint analysis (day 10), the vast majority of EBs were bright GFP^+ (Figure 4B) and contracting (Movie S1). On average, 62.9% ± 7.3% GFP^+ and 68.6% ± 8.7% $cTNT^+$ cells were observed in three individual bioreactor runs (53.9%–84.1%; Figure 4C and Table 1). When multiplied with total cell yields, on average, ~40 million CMs were observed per process (Figure 4C), whereby more than ~50 million CMs were generated in individual runs. Flow cytometry for sarcomeric markers α-actinin, MHC, and the cell surface marker SIRPα showed CM content of up 85% CMs at process endpoint (Figure 4E). This observation was further confirmed by immunofluorescence staining on EB sections or EB-derived, seeded cells displaying typical cross-striations (Figure 4D).

To substantiate the robustness of the C-perfusion-based differentiation, additional experiments were performed using hHSC_F1285T_iPS2 cells (Hartung et al., 2013). The CPA formation pattern of the hiPSC line (Figure S4A and S4B) was highly equivalent to the HES3 NKX2-$5^{eGFP/w}$ pattern (Figures 3A and 3B), resulting in an average aggregate size of 531.6 ± 35.3 nm before differentiation (day 0; Figure S4B). The subsequent cardiac differentiation led to homogeneously contracting EBs (Movie S2). On average, three independent runs revealed 53.2% ± 16.4% positivity for MHC, 58% ± 30.3% cTNT, and 51.2% ± 14.6% α-actinin (Figure S4C; range, 27.2%–83.5%; MHC, 27.7%–88.3% cTNT; Table 1).

Electrophysiological and Pharmacological Assessment Confirms Formation of Functional CMs, which Can Be Directly Applied for Tissue Engineering

Reactor-derived CMs were seeded onto MEAs to measure field potentials in the presence of chronotropic and arrhythmogenic drugs. Consistent with published data (Mandel et al., 2012), we have detected a positive chronotropic response by the beta-adrenergic agonist isoproterenol (Figure 5A). The class I antiarrhythmic compound quinidine, known to have complex interactions with ion channels, induced reversible spike amplitude reduction and prolonged the field potential duration (FPD) at 10 μM (Figure 5B), in line with recent findings (Braam et al., 2010; Harris et al., 2013). The class IV antiarrhythmic drug verapamil exerts its action by blocking calcium channels but is also a potent hERG blocker. The compound induced a dose-responsive negative chronotropic effect at 100 nM. This effect was more prominent at 300 nM and accompanied by a shortening of the FPD (Figure 5C), which most likely resulted from multiple channel blocks, thereby compensating the known hERG blocking effect, in agreement with clinical data (Braam et al., 2010).

Electrophysiological properties were further analyzed by whole-cell patch clamp recordings in the current clamp mode. Cells were classified according to the shape of their APs (Figure 5D). Thirty five of 41 cells (comprising 20 and 21 cells, respectively, derived from two independent differentiations) displayed ventricular-like APs. Six were classified as atrial-like (Figure 5E), whereas no AP patterns indicating nodal-like CMs were identified. The data were corroborated by comparing spontaneous versus evoked APs applying short depolarizing current steps (Figure S5).

Taken together, these pharmacological and electrophysiological results underscore that our method led to the formation of bona fide human CMs, displaying typical characteristics of early hPSC-derived cells (Navarrete et al., 2013).

Bioreactor-derived EBs were also used for the generation of BCTs. After matrix solidification, EBs formed synchronously contracting syncytia during the initial cultivation phase (days 0–4; data not shown). The addition of fibroblasts to the EB/collagen-I matrix was not essential to achieve tissue consolidation in contrast to a prior approach using genetically enriched EBs, which consisted of >99% CMs (Kensah et al., 2012). However, qualitative microscopic comparison of tissue formation on day 21 suggested slightly brighter NKX2.5-GFP intensity of constructs that

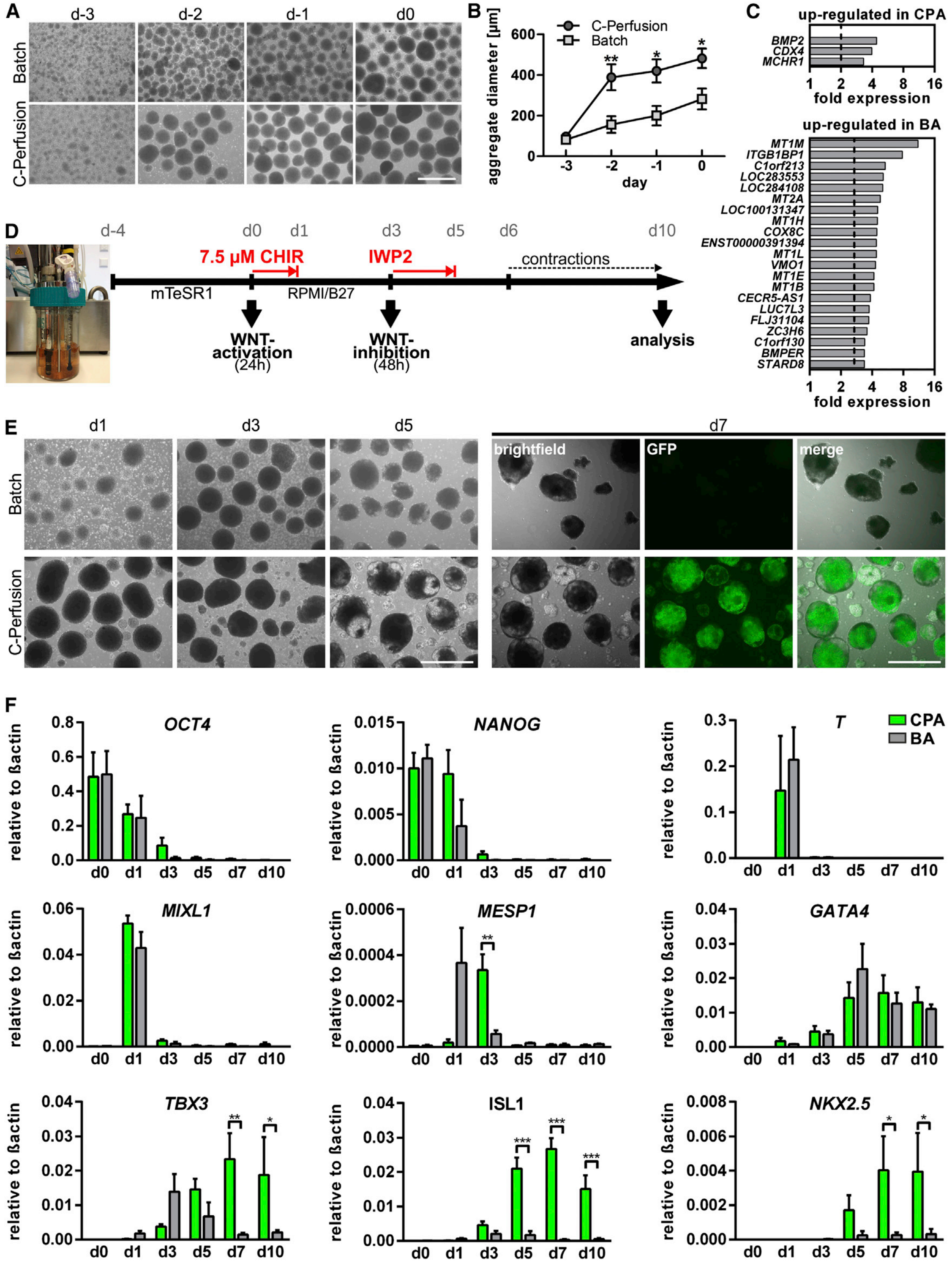

Figure 3. Feeding Strategy Determines the Differentiation Outcome in Stirred Bioreactors

(A) Representative aggregate images generated from batch (top) and C-perfusion (bottom). The scale bar represents 1 mm.

(B) Significantly larger spheres were observed at C-perfusion 24 hr postinoculation (n = 3 bioreactor runs, mean ± SEM).

(C) Upregulated genes in C-perfusion-derived CPAs (top) and batch-derived Bas (bottom) detected by microarray analysis. Gene lists represent the intersecting set of >2-fold upregulated and significantly regulated genes identified by RCUTAS and Qlucore Omics Explorer, respectively.

(legend continued on next page)

were prepared with the addition of human foreskin fibroblasts (with HFF; n = 5) compared with the EB-only group (without HFF, n = 4; exemplary depicted in Figure 5F), suggesting that HFF might support CMs persistence in respective constructs. Furthermore, BCTs generated with HFFs showed significantly higher contraction forces compared with the control group (1.59 ± 0.2 versus 0.4 ± 0.23 mN) and a more physiological Frank Starling mechanism, whereas no significant differences in passive forces were observed between these experimental groups (Figure 5G).

DISCUSSION

Focusing on critical culture parameters, we have developed and upscaled the production of hPSC-derived CMs in suspension culture and show their utility for tissue engineering.

Chemical Wnt pathway modulators were recently applied to direct cardiomyogenic differentiation of hPSCs seeded on matrigel or the synthetic matrix Synthemax (Gonzalez et al., 2011; Lian et al., 2012; Lian et al., 2013). Disregarding upscaling limitations of monolayer cultures, respective matrices might be a prerequisite for these protocols to work. By successful transition to free floating aggregates, we demonstrate that matrices and direct cell-to-substrate contacts are dispensable, stripping away another (costly) layer toward fully defined conditions.

In 2D, the generation of confluent monolayers ahead of differentiation was noted to be critical for efficient cardiomyogenesis (Lian et al., 2013). This suggests that the cell density, which impacts on cell-cell contacts and the concentration of paracrine factors, plays a role. Lian et al. (2013) thus provided cell numbers for monolayer inoculation, but the resulting cell densities at the induction of differentiation were not noted. In contrast to surface-attached cultures, cell density assessment and adjustment are straightforward in suspension. Particularly in stirred bioreactor scale, regular sampling allows close monitoring of cell counts and the assessment of other cell and aggregate properties, thereby ensuring good process definition and reproducibility. Here, we initiated differentiation when cultures reached a defined density of ~0.33×10^6 cells/ml. Although further investigations on the effect of cell density are required, this ensures improved cross-platform and intercell line comparability and provides a valuable reference point.

Keeping all other parameters constant, the concentration of the GSK3 inhibitor CHIR dictated the differentiation outcome in our screening platform; 7.5 μM CHIR was found to be optimal for cardiomyogenesis of hES3 NKX2.5-GFP cells in static and dynamic suspension culture and was also applicable to two independent hiPSC lines. In hESC monolayers, 10 μM CHIR was found to be optimal for inducing primitive streak markers, mesoderm, and ultimately cardiomyogenesis (Gonzalez et al., 2011), whereas 12 μM worked best in a related setup (Lian et al., 2012, 2013). Here CHIR concentrations >7.5 μM significantly reduced the CM content and overall cell yields, suggesting differential activity of GSK3 in suspension versus monolayer cultures. Alternatively, or in addition, the activity of other canonical Wnt pathway components and/or other pathways effecting cardiomyogenesis might differ in 3D versus 2D.

Numerous studies have suggested an impact of a defined sphere size on hPSC lineage differentiation. In microwell plates, for example, 1,000 hESCs per aggregate differentiated more efficiently into CMs compared with 100 or 4,000 hESCs per aggregate, respectively, whereby the size-dependent formation of definitive endoderm was found to trigger cardiac mesoderm induction by paracrine factors (Bauwens et al., 2011). In hydrogel microwells, cardiogenesis was enhanced in EBs of 450 μm diameter, whereas endothelial cell differentiation was increased at 150 μm, apparently due to EB size-dependent expression of noncanonical WNTs (Hwang et al., 2009). In simple, agarose-based microwells, Dahlmann et al. generated defined sphere batches ranging from 666 to 2,666 hESCs per aggregate (representing 185 to 270 μm mean diameter; J. Dahlmann, personal communication), respectively, followed by aggregate harvest and differentiation in dynamic suspension (Dahlmann et al., 2013). Although the initial aggregate size had an impact on hPSC growth kinetics and subsequently on overall cell and CM yields, the resulting CM content remained in a relative narrow range of ~40%–50% at all aggregate sizes tested (Dahlmann et al., 2013).

Despite the excellent experimental utility of culture platforms generating uniform aggregates or colony patterns, respective studies often ignore culture aspects such as the overall cell density or lack informative controls of mixed

(D) Scheme of the defined expansion/differentiation protocol in bioreactors.
(E) Microscopic assessment of differentiating aggregates/EBs from days 1 to 5 (left) and GFP fluorescence on day 7, which was discovered only upon differentiation of C-perfusion-derived cells (right). The scale bar represents 1 mm.
(F) Gene expression analysis by qRT-PCR for markers of pluripotency (*NANOG, OCT4*), primitive streak (*T-brachyury* and *MIXL1*), mesoderm (*MESP1*, *GATA4*, *TBX3*), and early cardiomyogenesis (*ISL1*, *NKX2.5*) comparing differentiation of BAs (gray columns) and CPAs (green) (n = 3 independent bioreactor runs each).
See also Figures S1–S3 and Movie S1.

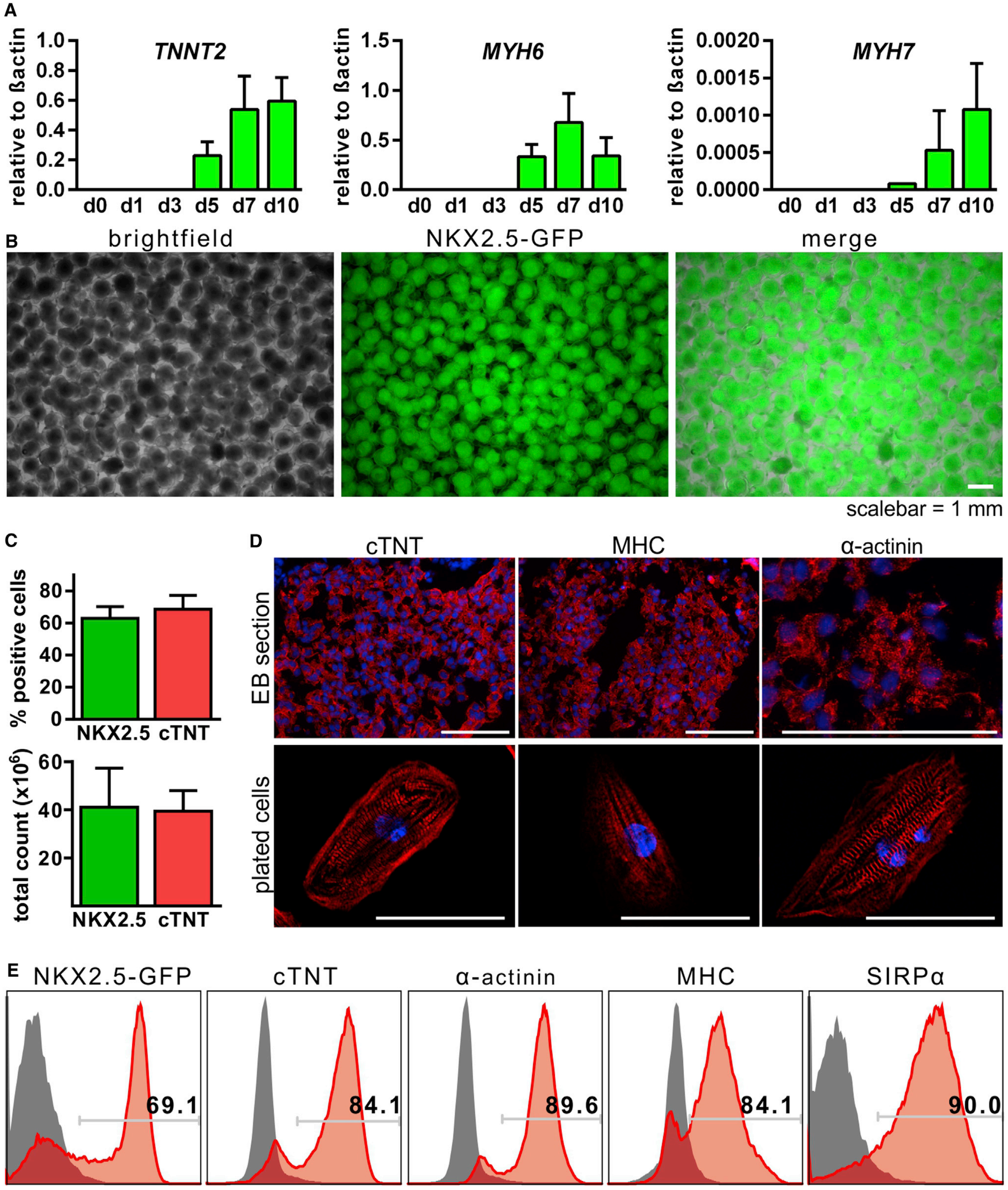

Figure 4. Characterization of Cardiomyogenesis in Cyclic Perfusion-Initiated Differentiations Revealed an Average Production of 40 × 10^6 CMs per Bioreactor Run, with >60% Average and up to ~85% CM Content

(A) Gene expression analysis by qRT-PCR for cardiac specific markers over time (n = 3 independent bioreactor runs).

(B) Microscopic assessment of NKX2.5-GFP transgenic HES3 on day 10 of differentiation shows homogenous GFP expression in nearly all EBs.

(legend continued on next page)

Table 1. Overview of CPA Differentiations

Run	Cell Line	Cells per Milliliter		Positive Cells (%)			
		Day 0	Day 10	NKX2.5	cTNT	MHC	α-Actinin
1	HES3 NKX2-5$^{eGFP/w}$	3.00×10^5	4.40×10^5	69.1	84.1	81.1	89.6
2	HES3 NKX2-5$^{eGFP/w}$	3.00×10^5	1.00×10^6	71.3	53.9	53.2	ND
3	HES3 NKX2-5$^{eGFP/w}$	3.00×10^5	4.00×10^5	50.8	67.8	66.8	67.3
4	hHSC_1285T_iPS2	3.00×10^5	8.88×10^5	NA	27.7	27.2	26.1
5	hHSC_1285T_iPS2	3.00×10^5	1.20×10^6	NA	88.3	83.5	76.7
6	hHSC_1285T_iPS2	3.00×10^5	1.25×10^6	NA	ND	48.8	50.7

NA, not applicable; ND, not determined.

sphere sizes. Comparing EB formation methods, such as random induction in suspension versus size-controlled spin EBs, Hong et al., (2010) noted that, despite a potential influence of the EB size, cell density-dependent, and (co) culture-dependent medium, conditioning dominantly defined hESC hematopoietic differentiation. Generating defined EBs by forced aggregation of mouse ESCs instructive clues on differentiation were rather induced by rotation speed-dependent hydrodynamic forces, whereas minor, if any, EB size-dependent effects were observed (Kinney et al., 2012).

In our static 12 wells, the average aggregate diameter was ~210 μm, representing a relatively large spread of ~100–400 μm in each well, typical of random aggregation. However, at 7.5 μM CHIR, aggregates of all shapes and sizes differentiated into contracting, GFP$^+$ EBs, whereas, at suboptimal CHIR concentrations, GFP expression was reduced or failed in respective wells, again without apparent aggregate/EBs size correlation. In stirred Erlenmeyer Flasks, the mean aggregate diameter was ~400 μm (~300–600 μm), and in bioreactors, at C-perfusion, it was ~450 μm (~350–600 μm), representing about double the mean diameter in 12 wells. These culture-dependent disparities were still compatible with efficient CM formation applying the same preoptimized differentiation protocol. In contrast, following batch expansion, cardiomyogenesis completely failed in repeated bioreactor runs, although the mean aggregate diameter of ~300 μm (~150–450 μm) was within the range of successful conditions.

Taken together, our cross-platform findings suggest that the initial aggregate size before induction of differentiation per se is not the dominant factor determining cardiac differentiation outcomes in suspension culture. Within a given size range, variations can be tolerated.

In the hPSC expansion phase, batch feeding was applied in static 12-well dishes and in stirred Erlenmeyer flasks, whereas in bioreactors, (cyclic) perfusion feeding led to successful cardiac differentiation. This demonstrates that different cell feeding protocols ahead of differentiation were compatible with subsequent cardiomyogenesis.

In the bioreactor setup the spatiotemporal pattern of aggregate development was the major determinant of subsequent differentiation results. We and others have shown that the pattern of PSC aggregation and further sphere development can be controlled well in stirred bioreactors by means of the inoculation density, the impeller design, and the stirring speed (Hunt et al., 2014; Olmer et al., 2012; Schroeder et al., 2005), including the on/off patterns applied in this study. In addition to the feeding-dependent differences of the culture milieu, the striking distinction was the continuous growth of initially small aggregates at batch, whereas at C-perfusion relative large CPAs were formed at 24 hr with a minor increase in size thereafter.

Although global profiling revealed rather uniform gene expression patterns typical of pluripotent cells under both expansion conditions, expression of the key morphogen BMP2 was highly upregulated in C-perfusion cultures. Modest BMP2 and BMP4 expression is suggested to act as an endogenous prodifferentiation signal in hESC, although these levels are insufficient to promote differentiation at pluripotent culture conditions (Teo et al., 2012). However, upon differentiation, the dominant role

(C) Relative (top) and total (bottom) numbers of NKX2.5-GFP- or cTNT-positive cells on day 10 of differentiation (n = 3 independent bioreactor runs).
(D) Immunofluorescent staining specific to sarcomeric proteins on HES-derived EB sections (top; the scale bar represents 100 μm) and iPS-derived, plated cells from EBs dissociated on day 10 (bottom; the scale bar represents 50 μm).
(E) Representative flow cytometry data of EB-derived cells.
See also Figure S4.

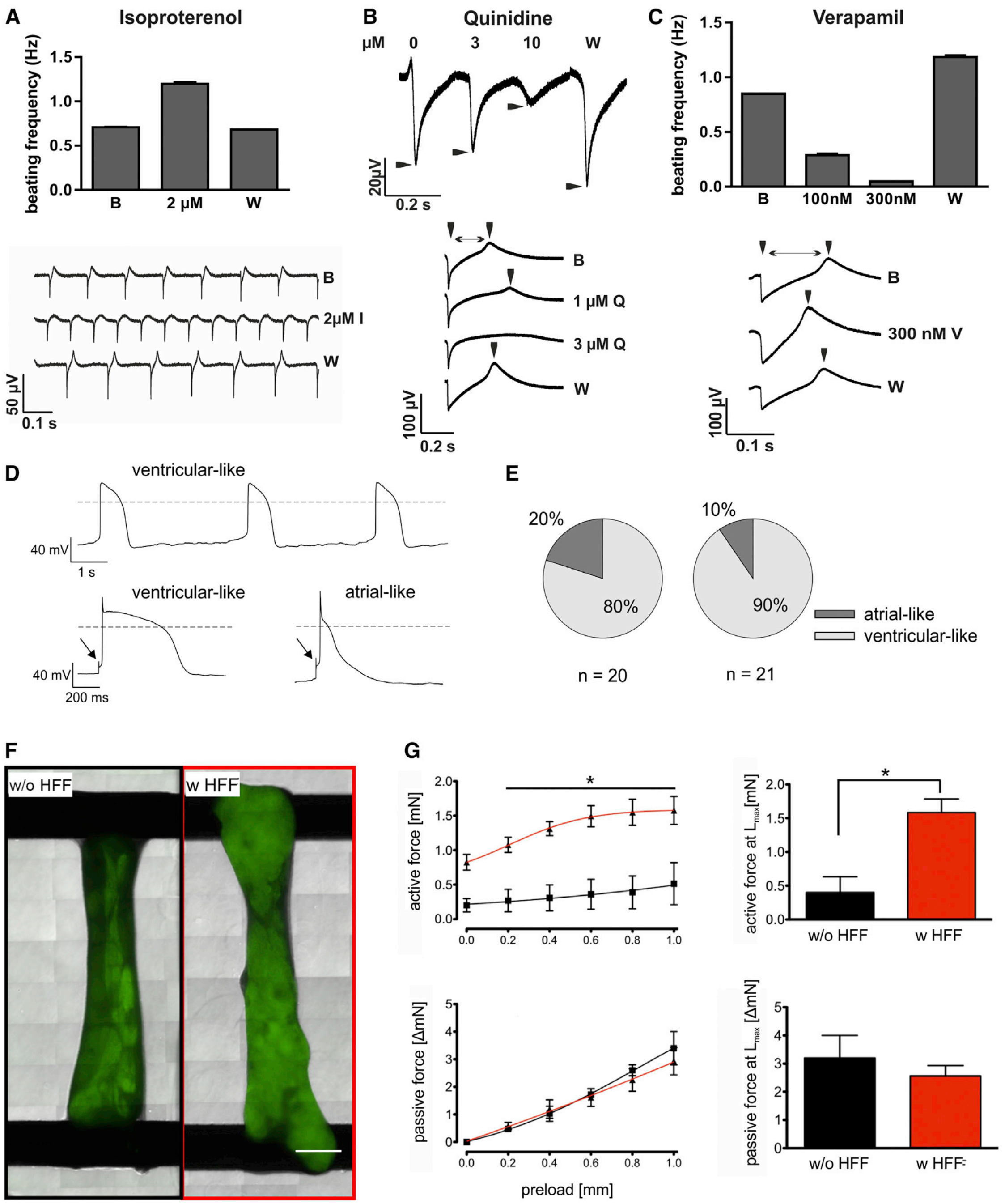

Figure 5. Electrophysiological and Pharmacological Characterization and Generation of BCT from Bioreactor-Derived EBs

(A–C) MEA-derived field potentials (FPs) revealed a positive chronotropic response by the beta-adrenergic agonist isoproterenol at 2 μM (A), prolonged FP duration at >1 μM quinidine (Q), and reduced spike amplitude at 10 μM (B), and a negative chronotropic response accompanied by a shortening of the FP duration in response to 300 nM verapamil (V) treatment (C). Effects were reversible by washout (W) (baseline [B]). n = 2 independent experiments.

(D) Representative recordings of a spontaneously active CM displaying a ventricular-like AP (top) and evoked APs representing ventricular- and atrial-like cells (bottom). Arrows in the bottom panels denote initial voltage responses to intracellular stimulation by short depolarizing current steps (left: 600 pA, 1 ms; right: 900 pA, 1 ms).

(E) Distribution of atrial- and ventricular-like cells derived from two differentiations.

(F) BCTs generated from EBs without (w/o, black lines/columns) or with (w, red) HFF on day 21. The scale bar represents 1 mm.

(legend continued on next page)

of BMP signaling on posterior primitive streak and subsequently on mesoderm formation in hPSCs was recently underscored (Loh et al., 2014). The upregulation of BMP2 as well as CDX4 (involved in anteroposterior axis specification regulated by Wnt activity; Hikasa et al., 2010) in CPAs might suggest the priming of these cells for mesoderm differentiation ahead of CHIR supplementation.

Loh et al. (2014) further noted the necessity to neutralize endogenous BMP to eliminate mesoderm induction of hPSCs. The upregulation of BMPER (BMP endothelial cell precursor derived regulator), an established antagonist of BMP2, BMP4, and BMP6 (Moser et al., 2003), in BAs (bottom panel in Figure 3C) might act as a neutralizing signal and, at least in part, explain the entire lack of cardiomyogenesis upon differentiation of these cells.

The control of cardiac differentiation by BMP signaling is well established (Laflamme and Murry, 2011). However, the role of autocrine/paracrine hPSC-derived BMP agonists and antagonists in modulating WNT pathway controlled differentiation is not well studied. Although future investigations on this are necessary, our data indicate the importance of this interplay.

However, primitive streak marker expression, including T and MIXL, was clearly present in BA-derived EBs after CHIR supplementation, suggesting that mesendodermal differentiation proceeds normally. Our data suggest that the temporal shift in the expression of key lineage transcription factors, such as MESP1 and TBX3, in CPA compared with BA cultures might underlie the divergence in differentiation outcomes.

We further found that the expression of 6 of 13 tested subtypes of the major metallothionein isoforms 1 and 2 (MT1, MT2) was upregulated in BAs. Since MTs bind to metal ions, a role of these proteins in hematopoietic cell proliferation and differentiation was suggested, but regulation of MTs expression by numerous stimuli, including oxidative stress (Takahashi, 2012), has been described. In mouse ESCs, upregulation of MT1 was discovered after addition of the p38 mitogen-activated protein kinase inhibitor PD169316 at LIF starvation-induced stress conditions (Duval et al., 2006). Overexpression of MT1 was notably sufficient to protect mESC from differentiation-induced apoptosis. To date, little is known about the functional role of MT in human PSCs, but we hypothesize that the feeding-induced fluctuation of the culture environment at batch conditions induces MT expression, as compared with more homogeneous conditions at C-perfusion. Although further investigation is required, metallothionein might thus present an interesting marker of stress response in hPSC culture. In this context, it is noteworthy that the stress-related genes HSPA1A and HSPA1B (HSP70 protein) were both found to be ~2-fold upregulated in BAs compared with CPAs (data not shown).

Following fundamental analysis of pharmacological and electrophysiological features of suspension-derived CMs, we have demonstrated direct applicability of bioreactor-produced EBs for tissue engineering. While BCT generation from undissociated, contracting spheres was recently shown, this required genetic enrichment of CMs before tissue formation (Kensah et al., 2012). On the other hand, enrichment to ~99% CM purity was incompatible with direct BCT generation, but required 15% fibroblast addition to remodel the initial collagen I matrix and hence support tissue formation (Kensah et al., 2012). Here, we show that the ~80% CM content resulting from our differentiation protocol was directly compatible with the production of functional tissues, suggesting that residual non-CMs within EBs provided structural support. A detailed assessment of the phenotype and features of respective non-CMs is currently in progress.

Taken together, we show that bioreactor-controlled programs of hPSC culture can be used to direct the subsequent fate of hPSCs on differentiation. As recently noted, it is now necessary to focus on the improvement of mass suspension culture for hPSC production and differentiation (Chen et al., 2014). Our study provides a substantial step along this path.

EXPERIMENTAL PROCEDURES

Cell Culture

HES3 NKX2-5$^{eGFP/w}$ (Elliott et al., 2011), hCBiPS2 (Haase et al., 2009), and hHSC_F1285T_iPS2 (Hartung et al., 2013) were maintained at standard conditions on MEFs. Before transition to suspension culture, cells were accutase-treated (PAA Laboratories) and seeded at 5×10^4 cell/cm^2 on Geltrex-coated flasks either in KnockOUT-SR medium (Life Technologies) conditioned with MEFs or in mTeSR1 (STEMCELL Technologies) supplemented with 10 μM Y-27632 for 24 hr. Medium was replaced daily, and cells were passaged twice per week.

For the inoculation of suspension culture, dissociation into single cells was performed by accutase treatment for 5 min at 37°C followed by dilution to 3.3×10^5 cells/ml in mTeSR1 plus 10 μM Y-27632 and seeding into 12-well suspension plates (Greiner-BioOne) or Erlenmeyer flasks (125 ml scale; VWR-International) in 1.5 ml/well or 20 ml/flask, respectively. Flasks were agitated at 75 rpm (orbital-shaker, Infors-HT).

(G) Force measurements of BCTs generated with HFF showed significantly higher active forces (L_{max}; top right) and a more physiological Frank Starling mechanism (top left) compared with BCTs without HFF, but no significant difference in passive forces (bottom left and right). n = 4–5 of independent BCTs.
See also Figure S5.

Stirred DASbox minibioreactors (DASGIP/Eppendorf) were inoculated as described previously (Olmer et al., 2012) (Supplemental Experimental Procedures). For subsequent expansion strategies, see the Results.

Differentiation of resulting aggregates was induced on day 0 (4 days after single cell inoculation) using CHIR99021 (synthesized by the Institute of Organic Chemistry, Leibniz University Hannover or purchased from Millipore) at indicated concentrations for 24 hr. On day 3, IWP2 (Tocris) was added at 5 μM for 48 hr. During the differentiation, cells were kept in RPMI1640 (Life Technologies) supplemented with B27 (minus insulin). Medium was entirely replaced on days 0 (+CHIR), 1, 3 (+IWP2 or IWR1), and 5 applying a volume of 3, 20, or 100 ml depending on the respective culture format, i.e., 12-well dish, Erlenmeyer flask, or stirred bioreactor. Aggregates were cultured in RPMI1640 supplemented with B27 from day 7 onward.

Aggregate or EB samples were monitored by light microscopy; images were captured (AxiovertA1; Zeiss) and processed in AxioVision (Zeiss) to define diameter and size distribution. Mean diameters represent the arithmetic average of 400–700 independent spheres. For cell seeding, samples were dissociated by 1 mg/ml collagenase B (Roche) for 15–60 min (progressing with differentiation) at 37°C (Maltsev et al., 1993; Zweigerdt et al., 2011).

For standard methods and prepublished techniques, see the Supplemental Experimental Procedures.

Statistics

Data are presented as mean ± SEM. Unless otherwise noted, statistical significance was calculated using Student's t tests. To take account of multiplicity in group comparisons, one- or two-way ANOVA followed by Bonferroni's posttest was conducted. Statistical significance was assigned as *$p < 0.05$, **$p < 0.01$, and ***$p < 0.001$.

SUPPLEMENTAL INFORMATION

Supplemental Information includes Supplemental Experimental Procedures, five figures, and two movies and can be found with this article online at http://dx.doi.org/10.1016/j.stemcr.2014.09.017.

ACKNOWLEDGMENTS

We thank T. Scheper for providing basic fibroblast growth factor, A. Kirschning and G. Dräger for providing Y-27632 and CHIR99021, A. Haase for providing the iPSC lines, E. Stanley and A. Elefanty for providing the hES-NKX2.5 cell line, and J. Dahlmann for the helpful discussion on microwell-generated aggregates. We thank N. McGuinness for critical reading of the manuscript. We also thank the RCU Transcriptomics of the Hannover Medical School for performing the microarray and their support in analyzing the data. This work was funded by Cluster of Excellence REBIRTH (DFG EXC62/1), German Ministry for Education and Science (BMBF; grant no. 13N12606), and StemBANCC (support from the Innovative Medicines Initiative joint undertaking under grant agreement n° 115439-2, resources of which are composed of financial contribution from the European Union [FP7/2007-2013] and EFPIA companies' in kind contribution).

Received: February 21, 2014
Revised: September 24, 2014
Accepted: September 25, 2014
Published: October 30, 2014

REFERENCES

Agarwal, S., Holton, K.L., and Lanza, R. (2008). Efficient differentiation of functional hepatocytes from human embryonic stem cells. Stem Cells *26*, 1117–1127.

Amit, M., Laevsky, I., Miropolsky, Y., Shariki, K., Peri, M., and Itskovitz-Eldor, J. (2011). Dynamic suspension culture for scalable expansion of undifferentiated human pluripotent stem cells. Nat. Protoc. *6*, 572–579.

Bauwens, C.L., Song, H., Thavandiran, N., Ungrin, M., Massé, S., Nanthakumar, K., Seguin, C., and Zandstra, P.W. (2011). Geometric control of cardiomyogenic induction in human pluripotent stem cells. Tissue Eng. Part A *17*, 1901–1909.

Bondue, A., Lapouge, G., Paulissen, C., Semeraro, C., Iacovino, M., Kyba, M., and Blanpain, C. (2008). Mesp1 acts as a master regulator of multipotent cardiovascular progenitor specification. Cell Stem Cell *3*, 69–84.

Braam, S.R., Tertoolen, L., van de Stolpe, A., Meyer, T., Passier, R., and Mummery, C.L. (2010). Prediction of drug-induced cardiotoxicity using human embryonic stem cell-derived cardiomyocytes. Stem Cell Res. (Amst.) *4*, 107–116.

Bulnes-Abundis, D., Carrillo-Cocom, L.M., Aráiz-Hernández, D., García-Ulloa, A., Granados-Pastor, M., Sánchez-Arreola, P.B., Murugappan, G., and Alvarez, M.M. (2013). A simple eccentric stirred tank mini-bioreactor: mixing characterization and mammalian cell culture experiments. Biotechnol. Bioeng. *110*, 1106–1118.

Carrondo, M.J., Alves, P.M., Carinhas, N., Glassey, J., Hesse, F., Merten, O.W., Micheletti, M., Noll, T., Oliveira, R., Reichl, U., et al. (2012). How can measurement, monitoring, modeling and control advance cell culture in industrial biotechnology? Biotechnol. J. *7*, 1522–1529.

Chen, K.G., Mallon, B.S., McKay, R.D., and Robey, P.G. (2014). Human pluripotent stem cell culture: considerations for maintenance, expansion, and therapeutics. Cell Stem Cell *14*, 13–26.

Couture, L.A. (2010). Scalable pluripotent stem cell culture. Nat. Biotechnol. *28*, 562–563.

Dahlmann, J., Kensah, G., Kempf, H., Skvorc, D., Gawol, A., Elliott, D.A., Dräger, G., Zweigerdt, R., Martin, U., and Gruh, I. (2013). The use of agarose microwells for scalable embryoid body formation and cardiac differentiation of human and murine pluripotent stem cells. Biomaterials *34*, 2463–2471.

David, R., Brenner, C., Stieber, J., Schwarz, F., Brunner, S., Vollmer, M., Mentele, E., Müller-Höcker, J., Kitajima, S., Lickert, H., et al. (2008). MesP1 drives vertebrate cardiovascular differentiation through Dkk-1-mediated blockade of Wnt-signalling. Nat. Cell Biol. *10*, 338–345.

Davis, R.P., Ng, E.S., Costa, M., Mossman, A.K., Sourris, K., Elefanty, A.G., and Stanley, E.G. (2008). Targeting a GFP reporter gene to the MIXL1 locus of human embryonic stem cells identifies human

primitive streak-like cells and enables isolation of primitive hematopoietic precursors. Blood *111*, 1876–1884.

Donndorf, P., Strauer, B.E., Haverich, A., and Steinhoff, G. (2013). Stem cell therapy for the treatment of acute myocardial infarction and chronic ischemic heart disease. Curr. Pharm. Biotechnol. *14*, 12–19.

Duval, D., Trouillas, M., Thibault, C., Dembelé, D., Diemunsch, F., Reinhardt, B., Mertz, A.L., Dierich, A., and Boeuf, H. (2006). Apoptosis and differentiation commitment: novel insights revealed by gene profiling studies in mouse embryonic stem cells. Cell Death Differ. *13*, 564–575.

Elliott, D.A., Braam, S.R., Koutsis, K., Ng, E.S., Jenny, R., Lagerqvist, E.L., Biben, C., Hatzistavrou, T., Hirst, C.E., Yu, Q.C., et al. (2011). NKX2-5(eGFP/w) hESCs for isolation of human cardiac progenitors and cardiomyocytes. Nat. Methods *8*, 1037–1040.

Esmailpour, T., and Huang, T. (2012). TBX3 promotes human embryonic stem cell proliferation and neuroepithelial differentiation in a differentiation stage-dependent manner. Stem Cells *30*, 2152–2163.

Fridley, K.M., Kinney, M.A., and McDevitt, T.C. (2012). Hydrodynamic modulation of pluripotent stem cells. Stem Cell Res Ther *3*, 45.

Gadue, P., Huber, T.L., Paddison, P.J., and Keller, G.M. (2006). Wnt and TGF-beta signaling are required for the induction of an in vitro model of primitive streak formation using embryonic stem cells. Proc. Natl. Acad. Sci. USA *103*, 16806–16811.

Gonzalez, R., Lee, J.W., and Schultz, P.G. (2011). Stepwise chemically induced cardiomyocyte specification of human embryonic stem cells. Angew. Chem. Int. Ed. Engl. *50*, 11181–11185.

Haase, A., Olmer, R., Schwanke, K., Wunderlich, S., Merkert, S., Hess, C., Zweigerdt, R., Gruh, I., Meyer, J., Wagner, S., et al. (2009). Generation of induced pluripotent stem cells from human cord blood. Cell Stem Cell *5*, 434–441.

Harris, K., Aylott, M., Cui, Y., Louttit, J.B., McMahon, N.C., and Sridhar, A. (2013). Comparison of electrophysiological data from human-induced pluripotent stem cell-derived cardiomyocytes to functional preclinical safety assays. Toxicol. Sci. *134*, 412–426.

Hartung, S., Schwanke, K., Haase, A., David, R., Franz, W.M., Martin, U., and Zweigerdt, R. (2013). Directing cardiomyogenic differentiation of human pluripotent stem cells by plasmid-based transient overexpression of cardiac transcription factors. Stem Cells Dev. *22*, 1112–1125.

Hikasa, H., Ezan, J., Itoh, K., Li, X., Klymkowsky, M.W., and Sokol, S.Y. (2010). Regulation of TCF3 by Wnt-dependent phosphorylation during vertebrate axis specification. Dev. Cell *19*, 521–532.

Hong, S.H., Werbowetski-Ogilvie, T., Ramos-Mejia, V., Lee, J.B., and Bhatia, M. (2010). Multiparameter comparisons of embryoid body differentiation toward human stem cell applications. Stem Cell Res. (Amst.) *5*, 120–130.

Hoogaars, W.M., Engel, A., Brons, J.F., Verkerk, A.O., de Lange, F.J., Wong, L.Y., Bakker, M.L., Clout, D.E., Wakker, V., Barnett, P., et al. (2007). Tbx3 controls the sinoatrial node gene program and imposes pacemaker function on the atria. Genes Dev. *21*, 1098–1112.

Hudson, J., Titmarsh, D., Hidalgo, A., Wolvetang, E., and Cooper-White, J. (2012). Primitive cardiac cells from human embryonic stem cells. Stem Cells Dev. *21*, 1513–1523.

Hunt, M.M., Meng, G., Rancourt, D.E., Gates, I.D., and Kallos, M.S. (2014). Factorial experimental design for the culture of human embryonic stem cells as aggregates in stirred suspension bioreactors reveals the potential for interaction effects between bioprocess parameters. Tissue Eng. Part C Methods *20*, 76–89.

Hwang, Y.S., Chung, B.G., Ortmann, D., Hattori, N., Moeller, H.C., and Khademhosseini, A. (2009). Microwell-mediated control of embryoid body size regulates embryonic stem cell fate via differential expression of WNT5a and WNT11. Proc. Natl. Acad. Sci. USA *106*, 16978–16983.

Kensah, G., Gruh, I., Viering, J., Schumann, H., Dahlmann, J., Meyer, H., Skvorc, D., Bär, A., Akhyari, P., Heisterkamp, A., et al. (2011). A novel miniaturized multimodal bioreactor for continuous in situ assessment of bioartificial cardiac tissue during stimulation and maturation. Tissue Eng. Part C Methods *17*, 463–473.

Kensah, G., Roa Lara, A., Dahlmann, J., Zweigerdt, R., Schwanke, K., Hegermann, J., Skvorc, D., Gawol, A., Azizian, A., Wagner, S., et al. (2012). Murine and human pluripotent stem cell-derived cardiac bodies form contractile myocardial tissue in vitro. Eur. Heart J. *34*, 1134–1146.

Kinney, M.A., Saeed, R., and McDevitt, T.C. (2012). Systematic analysis of embryonic stem cell differentiation in hydrodynamic environments with controlled embryoid body size. Integr. Biol. (Camb.) *4*, 641–650.

Laflamme, M.A., and Murry, C.E. (2011). Heart regeneration. Nature *473*, 326–335.

Lian, X., Hsiao, C., Wilson, G., Zhu, K., Hazeltine, L.B., Azarin, S.M., Raval, K.K., Zhang, J., Kamp, T.J., and Palecek, S.P. (2012). Robust cardiomyocyte differentiation from human pluripotent stem cells via temporal modulation of canonical Wnt signaling. Proc. Natl. Acad. Sci. USA *109*, E1848–E1857.

Lian, X., Zhang, J., Azarin, S.M., Zhu, K., Hazeltine, L.B., Bao, X., Hsiao, C., Kamp, T.J., and Palecek, S.P. (2013). Directed cardiomyocyte differentiation from human pluripotent stem cells by modulating Wnt/β-catenin signaling under fully defined conditions. Nat. Protoc. *8*, 162–175.

Loh, K.M., Ang, L.T., Zhang, J., Kumar, V., Ang, J., Auyeong, J.Q., Lee, K.L., Choo, S.H., Lim, C.Y., Nichane, M., et al. (2014). Efficient endoderm induction from human pluripotent stem cells by logically directing signals controlling lineage bifurcations. Cell Stem Cell *14*, 237–252.

Lui, K.O., Zangi, L., Silva, E.A., Bu, L., Sahara, M., Li, R.A., Mooney, D.J., and Chien, K.R. (2013). Driving vascular endothelial cell fate of human multipotent Isl1+ heart progenitors with VEGF modified mRNA. Cell Res. *23*, 1172–1186.

Lundy, S.D., Zhu, W.Z., Regnier, M., and Laflamme, M.A. (2013). Structural and functional maturation of cardiomyocytes derived from human pluripotent stem cells. Stem Cells Dev. *22*, 1991–2002.

Maltsev, V.A., Rohwedel, J., Hescheler, J., and Wobus, A.M. (1993). Embryonic stem cells differentiate in vitro into cardiomyocytes

representing sinusnodal, atrial and ventricular cell types. Mech. Dev. *44*, 41–50.

Mandel, Y., Weissman, A., Schick, R., Barad, L., Novak, A., Meiry, G., Goldberg, S., Lorber, A., Rosen, M.R., Itskovitz-Eldor, J., and Binah, O. (2012). Human embryonic and induced pluripotent stem cell-derived cardiomyocytes exhibit beat rate variability and power-law behavior. Circulation *125*, 883–893.

Minami, I., Yamada, K., Otsuji, T.G., Yamamoto, T., Shen, Y., Otsuka, S., Kadota, S., Morone, N., Barve, M., Asai, Y., et al. (2012). A small molecule that promotes cardiac differentiation of human pluripotent stem cells under defined, cytokine- and xeno-free conditions. Cell Rep. *2*, 1448–1460.

Moser, M., Binder, O., Wu, Y., Aitsebaomo, J., Ren, R., Bode, C., Bautch, V.L., Conlon, F.L., and Patterson, C. (2003). BMPER, a novel endothelial cell precursor-derived protein, antagonizes bone morphogenetic protein signaling and endothelial cell differentiation. Mol. Cell. Biol. *23*, 5664–5679.

Navarrete, E.G., Liang, P., Lan, F., Sanchez-Freire, V., Simmons, C., Gong, T., Sharma, A., Burridge, P.W., Patlolla, B., Lee, A.S., et al. (2013). Screening drug-induced arrhythmia using human induced pluripotent stem cell-derived cardiomyocytes and low-impedance microelectrode arrays. Circulation *128* (*Suppl 1*), S3–S13.

Olmer, R., Haase, A., Merkert, S., Cui, W., Palecek, J., Ran, C., Kirschning, A., Scheper, T., Glage, S., Miller, K., et al. (2010). Long term expansion of undifferentiated human iPS and ES cells in suspension culture using a defined medium. Stem Cell Res. *5*, 51–64.

Olmer, R., Lange, A., Selzer, S., Kasper, C., Haverich, A., Martin, U., and Zweigerdt, R. (2012). Suspension culture of human pluripotent stem cells in controlled, stirred bioreactors. Tissue Eng. Part C Methods *18*, 772–784.

Ren, Y., Lee, M.Y., Schliffke, S., Paavola, J., Amos, P.J., Ge, X., Ye, M., Zhu, S., Senyei, G., Lum, L., et al. (2011). Small molecule Wnt inhibitors enhance the efficiency of BMP-4-directed cardiac differentiation of human pluripotent stem cells. J. Mol. Cell. Cardiol. *51*, 280–287.

Schroeder, M., Niebruegge, S., Werner, A., Willbold, E., Burg, M., Ruediger, M., Field, L.J., Lehmann, J., and Zweigerdt, R. (2005). Differentiation and lineage selection of mouse embryonic stem cells in a stirred bench scale bioreactor with automated process control. Biotechnol. Bioeng. *92*, 920–933.

Sepulveda, J.L., Belaguli, N., Nigam, V., Chen, C.Y., Nemer, M., and Schwartz, R.J. (1998). GATA-4 and Nkx-2.5 coactivate Nkx-2 DNA binding targets: role for regulating early cardiac gene expression. Mol. Cell. Biol. *18*, 3405–3415.

Singh, H., Mok, P., Balakrishnan, T., Rahmat, S.N., and Zweigerdt, R. (2010). Up-scaling single cell-inoculated suspension culture of human embryonic stem cells. Stem Cell Res. *4*, 165–179.

Takahashi, S. (2012). Molecular functions of metallothionein and its role in hematological malignancies. J. Hematol. Oncol. *5*, 41.

Teo, A.K., Ali, Y., Wong, K.Y., Chipperfield, H., Sadasivam, A., Poobalan, Y., Tan, E.K., Wang, S.T., Abraham, S., Tsuneyoshi, N., et al. (2012). Activin and BMP4 synergistically promote formation of definitive endoderm in human embryonic stem cells. Stem Cells *30*, 631–642.

Weidgang, C.E., Russell, R., Tata, P.R., Kühl, S.J., Illing, A., Müller, M., Lin, Q., Brunner, C., Boeckers, T.M., Bauer, K., et al. (2013). TBX3 Directs Cell-Fate Decision toward Mesendoderm. Stem Cell Rep. *1*, 248–265.

Willems, E., Spiering, S., Davidovics, H., Lanier, M., Xia, Z., Dawson, M., Cashman, J., and Mercola, M. (2011). Small-molecule inhibitors of the Wnt pathway potently promote cardiomyocytes from human embryonic stem cell-derived mesoderm. Circ. Res. *109*, 360–364.

Zweigerdt, R. (2009). Large scale production of stem cells and their derivatives. Adv. Biochem. Eng. Biotechnol. *114*, 201–235.

Zweigerdt, R., Olmer, R., Singh, H., Haverich, A., and Martin, U. (2011). Scalable expansion of human pluripotent stem cells in suspension culture. Nat. Protoc. *6*, 689–700.

Stem Cell Reports, Volume *3*

Supplemental Information

Controlling Expansion and Cardiomyogenic Differentiation of Human Pluripotent Stem Cells in Scalable Suspension Culture

Henning Kempf, Ruth Olmer, Christina Kropp, Michael Rückert, Monica Jara-Avaca, Diana Robles-Diaz, Annika Franke, David A. Elliott, Daniel Wojciechowski, Martin Fischer, Angelica Roa Lara, George Kensah, Ina Gruh, Axel Haverich, Ulrich Martin, and Robert Zweigerdt

Supplemental Figures

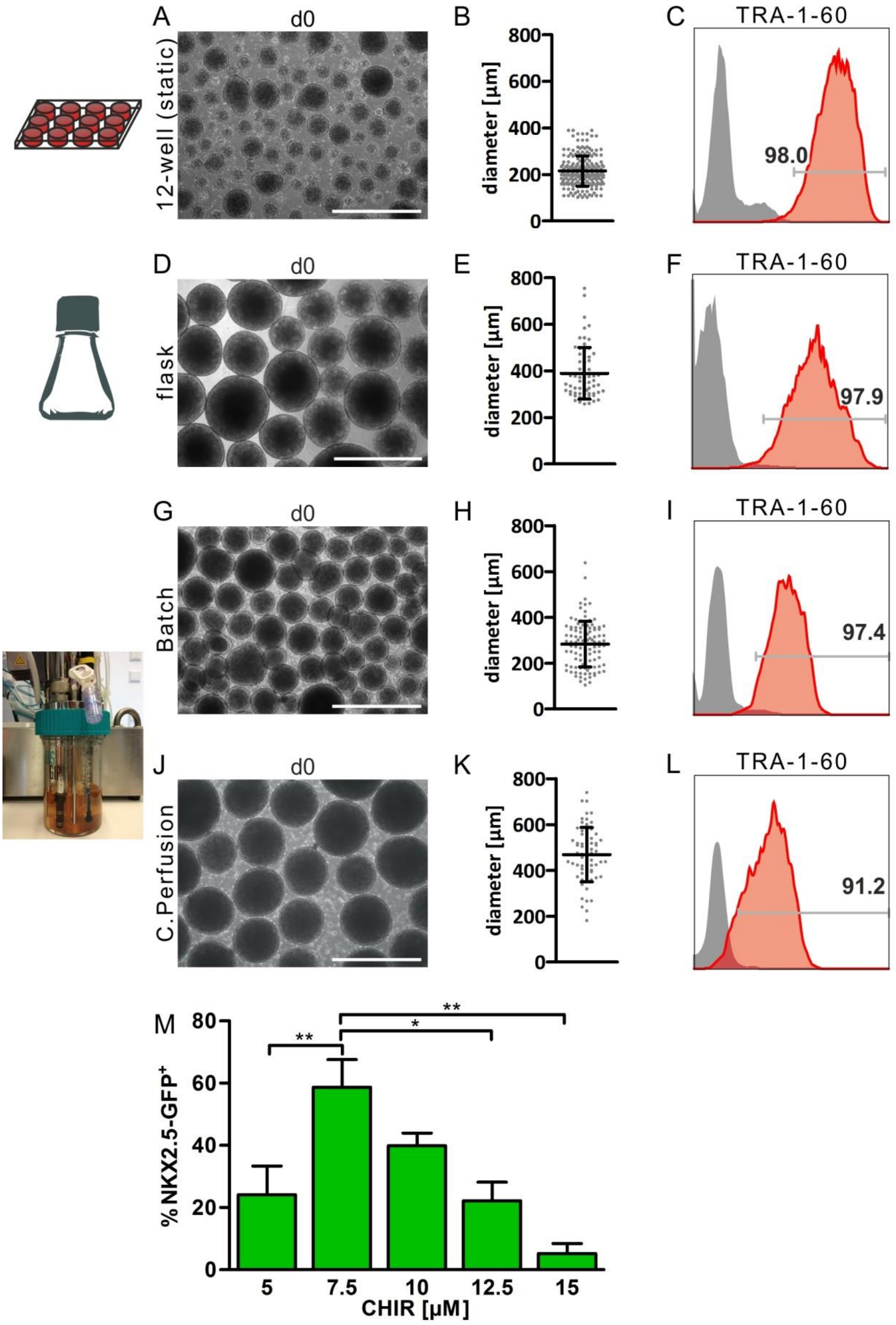

Figure S1. Characterization of aggregates before differentiation (day 0) generated in respective culture platforms, related to Figure 1, 2 and 3. Static culture **(A-C)**, agitated Erlenmeyer flask **(D-F)** and stirred bioreactors generated either by Batch **(G-I)** or C-Perfusion **(J-L)**, respectively. Representative microscopic images (left), quantification of aggregate diameters (middle; mean±SD) and flow cytometry data for TRA-1-60 positivity (right) are shown. Staining for TRA-1-60 (red) and respective IgM isotype controls (grey) are shown. Scale bars: 1 mm. **(M)** A similar pattern of NKX2.5-GFP was obtained using IWR1 instead of IWP2 and confirm 7.5 µM CHIR to induce highest level of GFP as quantified by flow cytometry on d10 of differentiation (n=3 independent experiments; mean±SEM).

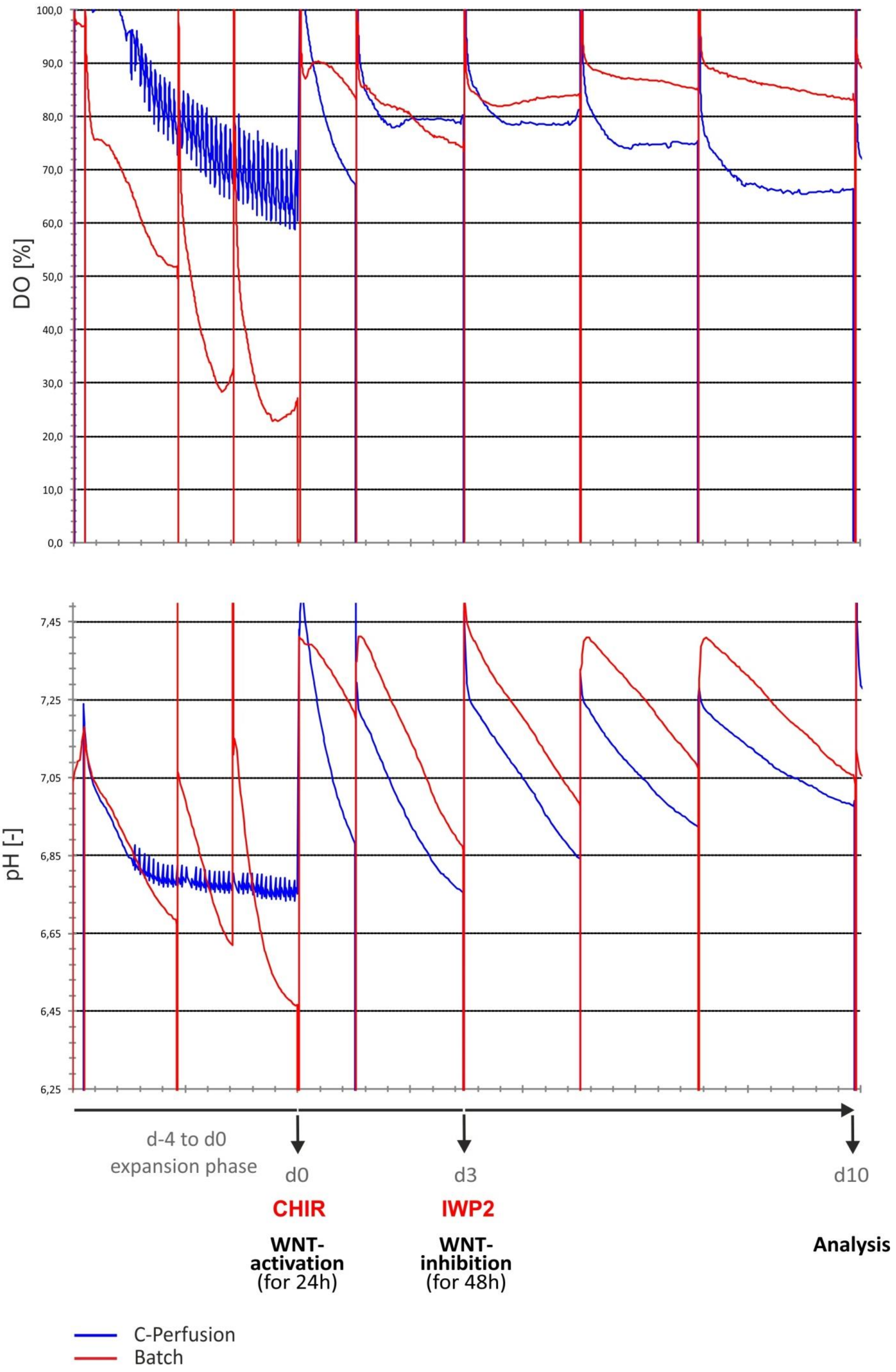

Figure S2. Online analysis of dissolved oxygen (DO) and pH during cell expansion and differentiation in stirred bioreactors, related to Figure 3. Representative DO levels (upper panel) and pH curves (lower panel) over time during the expansion phase (d-4 to d0) at C-Perfusion (blue line) or Batch feeding (red line) conditions and subsequent differentiation (d0 to d10) of respective cells.

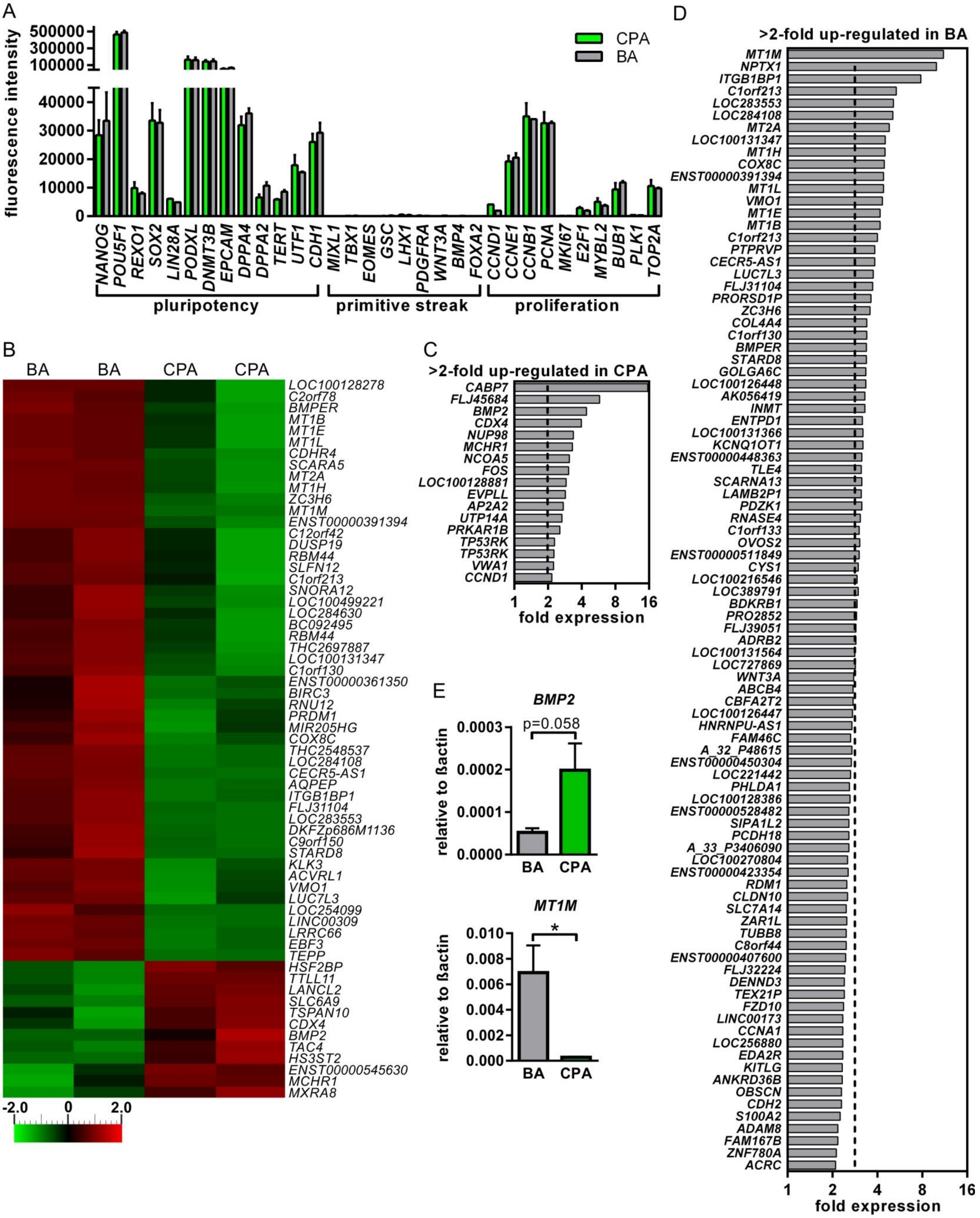

Figure S3. Microarray analysis comparing gene expression of bioreactor-derived aggregates (CPA vs. BA) prior to differentiation (day 0), related to Figure 3. (A) Absolute processed fluorescence intensity of markers for pluripotency, primitive streak formation and cell proliferation. **(B)** Heatmap displaying differentially expressed genes between BAs and CPAs detected by two group comparison using Qlucore Omics Explorer 3.0 ($p \leq 0.125$; $\sigma / \sigma_{max} = 0.35$). **(C, D)** Up-regulated genes in CPA and BA, respectively, which were identified using the RCUTAS filter tool (Intensity threshold 50; fold change cut-off 2.0). For further details see the experimental section. **(E)** qPCR of *BMP2* (upper panel) and *MT1M* (lower panel) confirming the results of microarray analysis (n = 3 of independent bioreactor runs; mean±SEM).

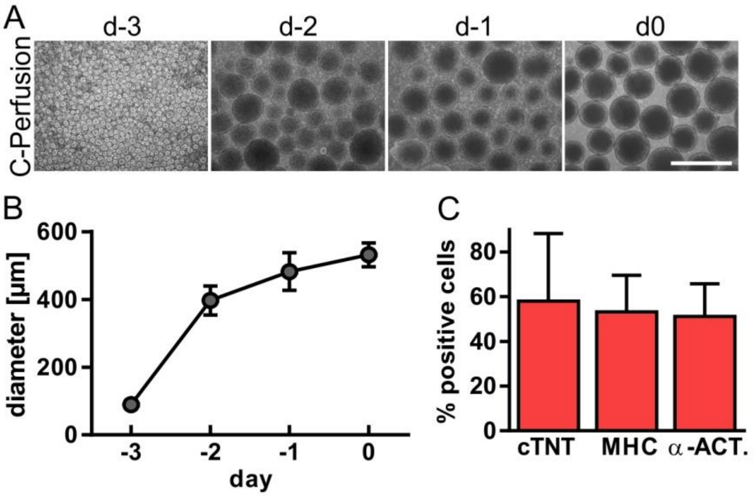

Figure S4. Characterization of Cyclic Perfusion bioreactor runs using the HSC_F1285T_iPS2 line confirms the robustness of the differentiation, related to Figure 4. (A) Representative images of aggregate formation in stirred bioreactors during C-Perfusion. Scale bar: 1mm **(B)** Quantification of aggregate diameters revealed formation of large spheres already 48h after culture inoculation (n=3 independent bioreactor runs, mean±SEM). **(C)** Differentiation efficiencies on d10 of differentiation (n=2 for cTNT, n=3 for MHC and α-ACTININ of independent bioreactor expansions; mean±SEM).

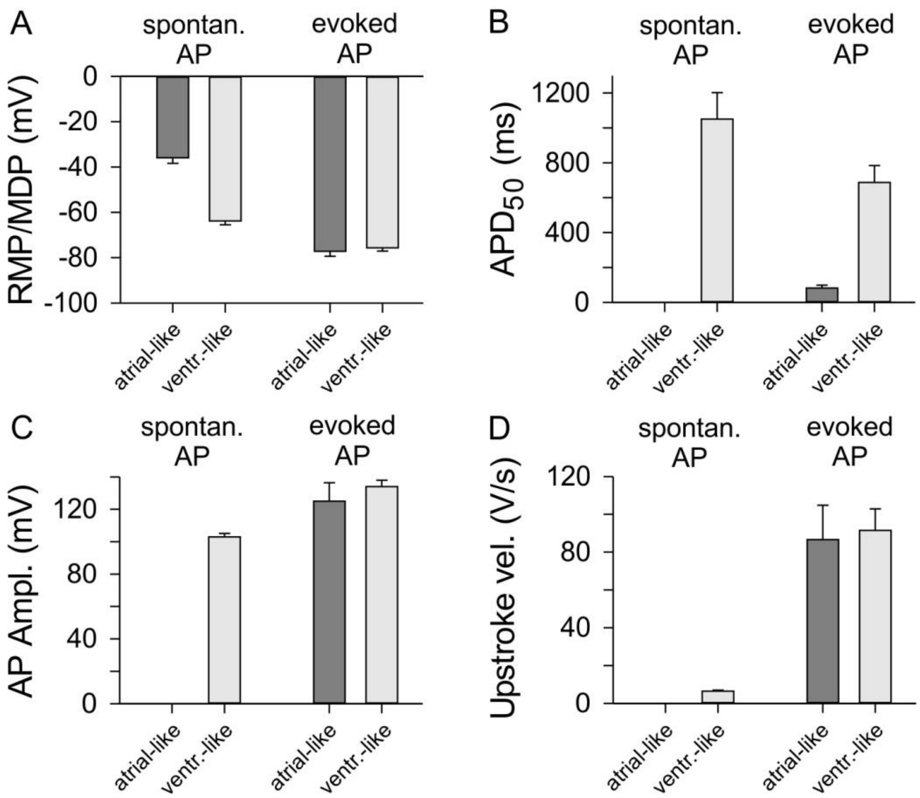

Figure S5. Electrophysiological properties of atrial-like (n=6) and ventricular-like (n=35) cardiomyocytes, related to Figure 5. Spontaneous action potentials (AP) are compared to APs evoked by intracellular injection of short depolarizing current steps (0.2 - 1.5 nA, 1 ms) after hyperpolarization of the plasma membrane to physiological resting potentials. **(A)** Atrial-like cells displayed small resting membrane potentials (RMP) and remained inactive without electrical stimulation. In contrast, 31 out of 35 ventricular-like cells were spontaneously active with a maximum diastolic potential (MDP) around -65 mV. Spontaneous activity declined with hyperpolarization to physiological resting potentials. **(B)** Evoked action potentials of atrial-like cells were shorter than those of ventricular-like cells (APD50: AP duration at 50% repolarization), whereas AP amplitudes and upstroke velocities were comparable **(C, D)**. Note that hyperpolarization to physiological resting conditions dramatically increased upstroke velocities of ventricular-like action potentials. Data represent mean±SEM.

Supplemental movie 1, related to Figure 3: Light and florescence microscopy of beating EBs of a CPA-based differentiation of the HES3 cell line.

Supplemental movie 2, related to Figure S4: Light microscopy of beating EBs of a CPA-based differentiation of the hHSC_1285T_iPS2 cell line.

Supplemental Experimental Procedures

Bioreactor calibration

Two-point calibration was applied for pH-probes. pO_2-probe calibration was conducted under process conditions: headspace-gassing with 3 L/h air/CO_2 (5%), 70 rpm stirring with 8 blade-pitched impeller, 37°C, 100 mL mTeSR1; reaching stable pO_2-values slope calibration was performed. For inoculation, 25 mL single cell suspension was added to achieve $5x10^5$ cells/mL in the final 125 mL culture volume.

Bioartificial cardiac tissue (BCT)

BCTs were prepared as described earlier (Kensah, 2013). In brief: ~60–80 bioreactor-derived EBs alone (w/o HFF) or with $1x10^5$ mitotically-inactivated human foreskin fibroblasts (w HFF) were mixed with a 0.8 mg/mL rat tail collagen I solution (R&D Systems) containing 10% matrigel (BD Biosciences) and poured into silicone molds for solidification in ~220 µL per tissue construct. Aiming at maturation and CM alignment, constructs were cultured and subjected to growing incremental static stretch (400 µm on d7, d11, d15, and d19) in a custom-made system (Kensah, 2011; Kensah, 2013). On d21 NKX2.5-GFP fluorescence was microscopically monitored to estimate group-dependent CM content (n=4 w/o HFF, n=5 w HFF). Force measurement: BCTs (n=3 w/o HFF, n=5 w HFF) were stretched by 200 µm increments allowing for preload-adjustment at each step and BCTs were electrically paced (25 V, 5 ms) five-times. The mean value of contraction force at each respective preload-position was recorded. L_{max} describes the length, i.e. preload position of individual tissue constructs, where maximum contraction forces were observed. Passive force determination: comparison of force sensor values at starting position versus values at respective preload step resulted in ΔmN. Additionally, passive force values at L_{max} for individual BCTs were determined.

Multielectrode Array (MEA)

Multielectrode array was performed as described previously (Wunderlich, 2012). In brief, bioreactor-derived EBs at d8-10 were dissociated and seeded at $0.5\text{-}1x10^6$ cells/array (60MEA200/30iR-Ti-gr, Multi Channel Systems; 0.1% gelatin, 4 µg/mL fibronectin-coated) in 1 mL IMDM (Invitrogen) containing 20% FCS (HyClone), 100 µM β-mercaptoethanol, 1% nonessential amino acids, 1% penicillin/streptomycin (Biochrom) and 10 µM Y-27632. All tested substances were purchased from Sigma-Aldrich, diluted in ultrapure water and added at concentrations as indicated. Recordings were performed and processed in MC-Rack (Multi Channel Systems) and visualized using GraphPad Prism.

Electrophysiology

CPA-derived cardiomyocytes were plated on gelatin/fibronectin-coated cover slips at differentiation day 10-13 and standard whole-cell patch clamp recordings were performed between day 19 and 27 of differentiation using an Axopatch 200B amplifier (Molecular Devices, Sunnyvale, CA). Pipettes were pulled from borosilicate glass (Harvard Apparatus, Holliston, MA) with resistances between 2 MΩ and 6 MΩ. The extracellular Thyrode solution contained (in mM): NaCl (140), KCl (5.4), CaCl2 (1.8), MgCl2 (1), HEPES (10), glucose (10), pH 7.4 (adjusted with NaOH). The intracellular solution contained (in mM): K-gluconate (120), MgCl2 (1), Mg-ATP (3), EGTA (10), HEPES (10), pH 7.2 (adjusted with KOH). Agar bridges were used to connect the amplifier and the pipette solution. Results were corrected for junction potentials calculated using the JPCalc software (Dr. P. Barry, University of South Wales, Sydney, Australia (Barry, 1994). Membrane potentials and spontaneous action potentials of single cardiomyocytes were recorded in the current clamp mode. Input resistances were determined from responses to small current steps and ranged from 500 MΩ to 5 GΩ. Cells with lower resistances were considered as leaky cells and rejected from evaluation. Cardiomyocytes were classified as atrial-like or ventricular-like based on the shape of their action potentials (AP). Resting membrane potential (RMP) or maximum diastolic potential (MDP), AP amplitude, AP duration at 50% repolarization (APD50) and upstroke velocity were determined. Cells with short plateau phase (< 200 ms) were classified as atrial-like cardiomyocytes. Electrophysiological properties were obtained from spontaneous firing and from evoked action potentials while cells were hyperpolarized to physiological resting potentials (70-80 mV). In the latter case action potentials were elicited by short depolarizing current steps (200 pA to 1.5 nA; 1 ms). Data were analyzed using Clampfit software (Axon Instruments, Union City, CA) and are presented as means±SEM.

Flow cytometry

For intracellular staining, $1.5x10^5$ cells were fixed/permeabilized according to manufacturer's instructions (Fix&Perm-kit; An der Grub). Antibodies specific to cardiac troponin T (1:200, clone 13-11, Thermo Scientific), α-ACTININ (1:800, clone EA-53, Sigma-Aldrich), myosin heavy chain (1:25, Hybridoma Bank; 1:2000, clone NOQ7.5.4D, Sigma-Aldrich), SIRPα (clone SE5A5; Biolegend), NKX2.5 (1:200; clone H-114; Santa Cruz) and respective isotype controls (Dako) were detected using appropriate Cy3-/Cy5-conjugated antibodies (1:200; Jackson Immunoresearch Laboratories). For surface staining, $1.5x10^6$ cells were incubated with anti-TRA-1-60-specific (1:100; mouse IgM, Abcam) and corresponding IgM isotype control (Dako) antibodies for 30 min at 4°C. After washing, signals were detected using Cy3-labeled donkey anti-mouse IgM (1:200; Jackson Immunoresearch Laboratories) on the Accuri C6 flow cytometer (BD Biosciences). Data were analyzed using FlowJo (Treestar).

Immunofluorescent staining

Dissociated cells were seeded on 0.1% gelatin-coated culture plates and fixed after 2 days with 4% paraformaldehyde, 15 min, RT. 10 µm cryosections were generated from liquid nitrogen-frozen aggregates/EBs embedded in Tissue-Tek (Sakura-Finetek) on HM 560 cryotome (Thermo Scientific). After blocking by Tris-buffered saline (5% donkey serum, 0.25% Triton X-100) cells or cryosections were incubated with primary/secondary antibodies listed in 'Flow cytometry', respectively. Nuclei were DAPI-stained and samples were analyzed using the Axio Observer A1 (Zeiss) or a DM IRB/TCS SP2 confocal microscope system (Leica).

Quantitative real-time PCR

Total RNA was prepared using RNeasy-Kit (Macherey-Nagel), reverse-transcribed with RevertAid™ H-Minus (Fermentas, Thermo Scientific) using random primers. qRT-PCR was performed using the Solaris qPCR Expression Assays (Thermo Scientific); expression levels of target genes were normalized to β-Actin levels.

Microarray analysis

The microarray has been performed by use of a refined version of the Whole Human Genome Oligo Microarray 4x44K v2 (Design ID 026652, Agilent Technologies), called '026652AsQuintuplicatesOn180k' developed in the Research Core Unit Transcriptomics of Hannover Medical School (design ID 054261) covering roughly 26000 human transcripts. Microarray design was defined at Agilent's eArray portal using an mRNA expression 4x180k design format as template. All non-control probes of design ID 026652 have been determined to be printed five times onto one 180k Microarray (on-chip quintuplicates). Control probes required for proper Feature Extraction software algorithms were determined and placed automatically by eArray using recommended default settings. Synthesis of Cy3-labeled cRNA was performed with the 'Quick Amp Labeling kit, one color' (#5190-0442, Agilent Technologies) according to the manufacturer's recommendations. cRNA fragmentation, hybridization and washing steps were also carried-out exactly as recommended in the 'One-Color Microarray-Based Gene Expression Analysis Protocol V5.7'. 1650ng of each labeled cRNA population were used for hybridization. Slides were scanned on the Agilent Micro Array Scanner G2565CA (pixel resolution 3 µm, bit depth 20). Data extraction was performed with the 'Feature Extraction Software V10.7.3.1' using a modified version of the recommended default extraction protocol file 'GE1_107_Sep09.xml' in which the minimal number of replicates to calculate Population Outliers was set to 5.

To identify >2-fold up-regulated genes the processed intensity values of the green channel, 'gProcessedSignal' (gPS) were normalized by global linear scaling: All gPS values of one

sample were multiplied by an array-specific scaling factor. This factor was calculated by dividing a ‘reference 75th Percentile value’ (set as 1500 for the whole series) by the 75th Percentile value of the particular Microarray (‘Array I’ in the formula shown below). Accordingly, normalized gPS values for all samples (microarray data sets) were calculated by the following formula:

$$normalized\ gPS_{Array\ i} = gPS_{Array\ i} \times (1500 / 75^{th}\ Percentile_{Array\ i})$$

Measurements of on-chip replicates (quintuplicates) were averaged using the arithmetic mean of normalized gPS values to retrieve one resulting value per probe and sample. Features that were marked as Outliers by the Feature Extraction software were excluded from averaging.

A lower intensity threshold (surrogate value) was defined as 1% of the reference 75th Percentile value (= 15). All of those normalized gPS values that fell below this intensity border, were substituted by the respective surrogate value of 15. Differentially expressed genes were identified by filtering >2-fold up-regulated genes (Intensity threshold 50; fold change cutoff 2.0) applying the RCUTAS filter tool (Research Core Unit Transcriptomics of Hannover Medical School). Results were ranked by fold change and displayed in a diagram.

Alternatively, extracted data were loaded into Qlucore Omics Explorer 3.0 (Qlucore AB, Lund, Sweden) applying default settings including normalization and baseline transformation. A two group comparison ($p \leq 0.125$; $\sigma / \sigma_{max} = 0.35$) was performed and visualized in a hierarchically clustered heatmap. Microarray data are available in the ArrayExpress database (www.ebi.ac.uk/arrayexpress) under accession number E-MTAB-2814.

Supplemental References

Barry, P. H. (1994). JPCalc, a software package for calculating liquid junction potential corrections in patch-clamp, intracellular, epithelial and bilayer measurements and for correcting junction potential measurements. J Neurosci Methods 51, 107-116.

Kensah, G., et al. (2011). A novel miniaturized multimodal bioreactor for continuous in situ assessment of bioartificial cardiac tissue during stimulation and maturation. Tissue engineering. Part C, Methods 17, 463-473.

Kensah, G., et al. (2013). Murine and human pluripotent stem cell-derived cardiac bodies form contractile myocardial tissue in vitro. Eur Heart J 34, 1134-1146.

Wunderlich, S., et al. (2012). Induction of pluripotent stem cells from a cynomolgus monkey using a polycistronic simian immunodeficiency virus-based vector, differentiation toward functional cardiomyocytes, and generation of stably expressing reporter lines. Cell Reprogram 14, 471-484.
